# Analysis of the Major Investment Object by Using a Novel Approach Based on Neutrosophic Information

**DOI:** 10.1155/2022/2092313

**Published:** 2022-01-10

**Authors:** Muhammad Umar Farooq, Rukhshanda Anjum, Abdul Gaffar, Huma Bashir, Naziha Al-Aidroos, Ahmed Alsanad

**Affiliations:** ^1^Department of Business Studies, Namal University, Mianwali 42250, Pakistan; ^2^Department of Mathematics and Statistics, University of Lahore, Lahore, Pakistan; ^3^Department of Mathematics, Ghazi University, Dera Ghazi Khan, Pakistan; ^4^Department of Basic Science and Humanities, Bahauddin Zakarya University, Multan, Pakistan; ^5^Computer Science Department, College of Computers and Information Technology, Hadhramout University, Hadhramout, Yemen; ^6^STC's Artificial Intelligence Chair, Department of Information Systems, College of Computer and Information Sciences, King Saud University, Riyadh 11543, Saudi Arabia

## Abstract

Neutrosophic set (NS) is an extensively used framework whenever the imprecision and uncertainty of an event is described based on three possible aspects. The association, neutral, and nonassociation degrees are the three unique aspects of an NS. More importantly, these degrees are independent which is a great plus point. On the contrary, neutrosophic graphs (NGs) and single-valued NGs (SVNGs) are applicable to deal with events that contain bulks of information. However, the concept of degrees in NGs is a handful tool for solving the problems of decision-making (DM), pattern recognition, social network, and communication network. This manuscript develops various forms of edge irregular SVNG (EISVNG), highly edge irregular SVNG (HEISVNG), strongly (EISVNG), strongly (ETISVNG), and edge irregularity on a cycle and a path in SVNGs. All these novel notions are supported by definitions, theorems, mathematical proofs, and illustrative examples. Moreover, two types of DM problems are modelled using the proposed framework. Furthermore, the computational processes are used to confirm the validity of the proposed graphs. Furthermore, the results approve that the decision-making problems can be addressed by the edge irregular neutrosophic graphical structures. In addition, the comparison between proposed and the existing methodologies is carried out.

## 1. Introduction

The theory of fuzzy sets (FSs) is one of the communalized notions of classical set theory. There are merely two prospects of a statement in classical set theory; the statement/event is either true or not. However, there are many statements that cannot be dealt with only these two prospects. FSs can be accurately employed to manage such statements that have variable values. Zadeh [1] developed the concept of FSs to manage the issues with uncertainties. FS theory has an important role in complicated process that could not simply categorized by classical set theory. Some years later, Atanassov [2] suggested the concept of intuitionistic FS (IFS) as a communalization of FS. Additionally, he also gives a novel element which demonstrates the falsity membership grade in the description of FS. The notion of IFS is more significant in addition to exhaustive because of truth membership grade and falsity membership grade, in which the indeterminacy membership degree of IFS is its hesitation membership grade. To some extent, both the truth and falsity membership degrees are independent from each other with the condition that the summation of both these degrees does not exceed one. By joining the nonstandard analysis, Smarandache [3] developed the notion of neutrosophic sets (NS).

In mathematics, NS is an instrument which is the generalization of classical set theory that is used to handle practical issues consisting of imprecise, indeterminate and varying information. Like the theories of FSs and IFSs, the theory of NSs is beneficial in several fields, such as topology, medicines, decision-making (DM) problems, and in many others practical issues. To manage NS more easily with daily life problems, Wang et al. [4] established the concepts of single-valued NSs (SVNS). An SVNS has three elements: truth, indeterminacy, and falsity membership degrees. These degrees are independent in an SVNS, and their values are enclosed in the standard unit interval [0, 1]. The SVNS is indeed an oversimplification of an IFS. The SVNS has been a very significant research topic recently, and several researchers have considered SVNS in their works [5–9]. Other related works, such as, Majumdar and Samanta [10], examined the entropy and similarity of SVNS. Correlation coefficients of SVNS were suggested by Ye [11, 12] and utilized it to SVN-DM problems.

Apart from that, the idea of graphs can be related to NS. Graph theory has turned out to be an influential framework to model and solve the joint problems that occur in many fields, such as mathematics, engineering, and computer sciences. An SVN graph (SVNG) has many characteristics which are the origin of various techniques that are employed in modern mathematics as it is the generalization of graphs. A lot of studies on FS, fuzzy graphs (FGs), and intuitionistic FGs (IFGs) [13–21] have been explored and every single one have considered the set of vertices and the set of edges as FSs and/or IFSs. However, the FG and IFG are unsuccessful when the relations between nodes (or vertices) in problems are not determined or not recognised. For this reason, Smarandache [3] introduced four major classes of the neutrosophic graphs (NGs). Two of these are built on literal indeterminacy, i.e., NGs are I-edge NG and I-vertex NG. The vast range of applications in decision-making problems made the NGs the hot topic for the researchers of the field. Since then, many attempts have been made to extend the notion of NGs. The work of Broumi et al. [22] stands alone, which is the introduction of a novel concept of SVNG. Besides that, Mohanta et al. [23] described the types of products of NGs and neutrosophic algebraic structures. Ramia et al. [24] defined the ideas of complimentary domination in SVNGs. The notion of operations of SVNG and interval-valued SVNG are discussed in the literature, see [25]. Abu Saleem [26] worked on the neutrosophic folding and a neutrosophic retraction on a SVNG. Lu and Ye [27] discussed SVN hybrid arithmetic and geometric aggregation operators. Lately, Shahzadi et al. [28] presented an application that carried out a medical diagnosis by using the concepts of SVNS.

However, the literature has great capacity when comes to the SVNGs and the types of their edges. Henceforth, this study intends to define the concepts of edge irregular SVNG and totally edge irregular SVNG. In addition, the path and cycle of an edge irregular SVNG will also be established. Instead of considering a general NG in which edges and vertices must be considered, our proposed works depart more formally from the three degree aspects of NG to interval three degree aspects of SVNG. Moreover, the proposed notions of SVNGs are applied to a couple of decision-making problems. The first problem is to select the best company among a collection of companies. To this end, the weighted averaging and weighted geometric aggregation operators were used as tool for the solution. While the second problem, which was targeted to select the best combination of subjects for a student of high school, was modelled and solved by the idea of edges in SVNGs. In order to provide strength to our study, we carried out a detailed comparison between the proposed framework and other contenders in the field. The experiments verified the validity of our method. The benefits of the proposed framework are as follows: (i) it is capable of modelling a complex situation, (ii) it can handle the events by describing three degrees, i.e., association, neutral, and nonassociation degrees, (iii) the decision maker can independently assign values to the degrees, and (iv) there is no constraints and limitations of these structures. Considering these benefits, we chose the SVNGs for our study.

In Section 2, some basic definitions are given which provide some base to construct further ideas. In Section 3, edge regular and highly edge regular SVNGs are defined.  Section 4 defines the strong edge irregular SVNGs and strong edge totally irregular SVNGs. In Section 5, the edge irregularity is discussed on a path and on a cycle in SVNGs. Then, the applications of the proposed concepts are presented in Section 6. Section 6 also contains the comparison of our method with the other methods. And finally, the concluding remarks are given in Section 7.

## 2. Preliminaries

Some basic definitions related to our graphical work such as IFG, SVNG, and degree of SVNG are presented in this section. Some examples are also presented to illustrate the notions.

**Definition 1 (see [14]).**

A pair $G=\left(\tilde{A},\text{Ê},A,B\right)$ is called IFG, where $\tilde{V}=\left\{{\tilde{V}}_{1},{\tilde{V}}_{2},{\tilde{V}}_{3},\ldots ,{\tilde{V}}_{n}\right\}$, $\text{ Ê}\subseteq \tilde{V}\times \tilde{V},$*A*=(*T*_1_, *L*_1_) is an IFS on $\tilde{V}$, and $B=\left(T_{2},L_{2}\right):\tilde{V}\times \tilde{V}⟶\left[0,1\right]\times \left[0,1\right]$ such that $T_{2}\left({\tilde{V}}_{i},{\tilde{V}}_{j}\right)\leq \min\left[T_{1}\left({\tilde{V}}_{i}\right),T_{1}\left({\tilde{V}}_{j}\right)\;\right]$ and $L_{2}\left({\tilde{V}}_{i},{\tilde{V}}_{j}\right)\leq \max\left[L_{1}\left(V_{i}\right),L_{1}\left(V_{j}\right)\;\right]$ with the condition $0\leq T_{2}\left({\tilde{V}}_{i},{\tilde{V}}_{j}\right)+L_{2}\left({\tilde{V}}_{i},{\tilde{V}}_{j}\right)\leq 1$, for all $\left({\tilde{V}}_{i},{\tilde{V}}_{j}\right)\in \text{Ê}.$

**Example 1 .**

**Figure 1:**
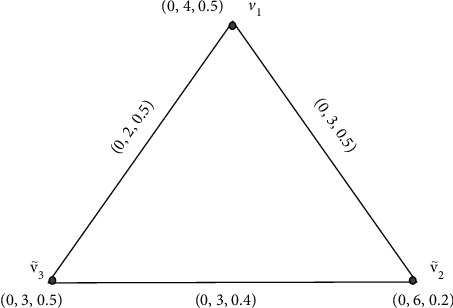
Intuitionistic fuzzy graph.

Let $\overset{\cdot }{G}=\left(\tilde{V},\text{Ê}\right)$ be an IFG, where $\tilde{V}$ is the collection of vertices and Ê is the collection of edges.  Figure 1 shows an IFG.

**Definition 2 (see [4]).**

A pair $G=\left(\tilde{V},\text{Ê},A,B\right)$ is known as SVNG, where $\tilde{V}=\left\{{\tilde{V}}_{1},{\tilde{V}}_{2},{\tilde{V}}_{3},\ldots ,{\tilde{V}}_{n}\right\}$, $\text{ Ê}\subseteq \tilde{V}\times \tilde{V},$$A=\left(T_{1},L_{1},{\dot{F}}_{1}\right)$ is an SVNS on $\tilde{V}$, and $B=\left(T_{2},L_{2},{\dot{F}}_{2}\right):\;\tilde{V}\times \tilde{V}\times \tilde{V}⟶\left[0,1\right]\times \left[0,1\right]\times \left[0,1\right]$ such that $T_{2}\left({\tilde{V}}_{i},{\tilde{V}}_{j}\right)\leq \min\left(T_{1}\left({\tilde{V}}_{i}\right),T_{1}\left({\tilde{V}}_{j}\right)\;\right],$$L_{2}\left({\tilde{V}}_{i},{\tilde{V}}_{j}\right)\geq \max\left(L_{1}\left(V_{i}\right),L_{1}\left(V_{j}\right)\;\right]\;$, and *F*_2_(*V*_*i*_, *V*_*j*_) ≥ max[*F*_1_(*V*_*i*_), *F*_1_(*V*_*j*_)] with the condition $0\leq T_{2}\left({\tilde{V}}_{i},{\tilde{V}}_{j}\right)+L_{2}\left({\tilde{V}}_{i},{\tilde{V}}_{j}\right)+{\dot{F}}_{2}\left({\tilde{V}}_{i},{\tilde{V}}_{j}\right)\leq 3$, for all (*V*_*i*_, *V*_*j*_) ∈ Ê.

**Example 2 .**

**Figure 2:**
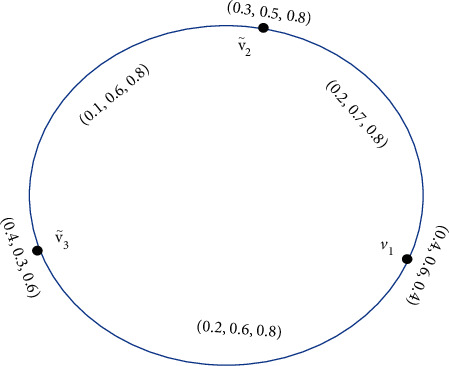
Single-valued neutrosophic graph.

An SVNG is shown in Figure 2.

**Definition 3 (see [4]).**

A pair $G=\left(\tilde{V},\text{Ê},A,B\right)$ is called strong SVNG, where $\tilde{V}=\left\{{\tilde{V}}_{1},{\tilde{V}}_{2},{\tilde{V}}_{3},\ldots ,{\tilde{V}}_{n}\right\}$, $\text{ Ê}\subseteq \tilde{V}\times \tilde{V},$*A*=(*T*_1_, *L*_1_, *F*_1_) is an SVNS on $\tilde{V}$, and $B=\left(T_{2},L_{2},F_{2}\right):\;\tilde{V}\times \tilde{V}\times \tilde{V}⟶\left[0,1\right]\times \left[0,1\right]\times \left[0,1\right]$ such that $T_{2}\left({\tilde{V}}_{i},{\tilde{V}}_{j}\right)=\min\left[T_{1}\left({\tilde{V}}_{i}\right),T_{1}\left({\tilde{V}}_{j}\right)\;\right]$, $L_{2}\left({\tilde{V}}_{i},{\tilde{V}}_{j}\right)=\max\left[L_{1}\left({\tilde{V}}_{i}\right),L_{1}\left({\tilde{V}}_{j}\right)\;\right]$, and $\;F_{2}\left({\tilde{V}}_{i},{\tilde{V}}_{j}\right)=\;\max\left[{\dot{F}}_{1}\left({\tilde{V}}_{i}\right),{\dot{F}}_{1}\left({\tilde{V}}_{j}\right)\right]$ with the condition $0\leq T_{2}\left({\tilde{V}}_{i},{\tilde{V}}_{j}\right)+L_{2}\left({\tilde{V}}_{i},{\tilde{V}}_{j}\right)+{\dot{F}}_{2}\left({\tilde{V}}_{i},{\tilde{V}}_{j}\right)\leq 3$, for all $\left({\tilde{V}}_{i},{\tilde{V}}_{j}\right)\in \text{Ê}.$

**Definition 4 (see [4]).**

The degree of a vertex in a SVNG $G=\left(\tilde{V},\text{Ê},A,B\right)$ is denoted and defined by $\deg\left(\;\tilde{V}\right)=\left(\deg_{t}\left(\tilde{V}\right),\deg_{l}\left(\tilde{V}\right),\deg_{\dot{F}}\left(\tilde{V}\right)\right)\;$, where $\;\deg_{T}\left(\tilde{V}\right)=\sum_{{\tilde{V}}_{i}\neq {\tilde{V}}_{j}}T_{B}\left({\tilde{V}}_{i},{\tilde{V}}_{j}\right)$, $\deg_{L}\left(\tilde{V}\right)=\sum_{{\tilde{V}}_{i}\neq {\tilde{V}}_{j}}L_{B}\left({\tilde{V}}_{i},{\tilde{V}}_{j}\right)$, and $\deg_{\dot{F}}\left(\tilde{V}\right)=\sum_{{\tilde{V}}_{i}\neq {\tilde{V}}_{j}}{\dot{F}}_{B}\left({\tilde{V}}_{i},{\tilde{V}}_{j}\right)$. Here, $\deg_{\underline{T}}\left(\tilde{V}\right)$ denotes the membership degree, $\deg_{L}\left(\tilde{V}\right)$ denotes the indeterminacy degree, and $\deg_{\dot{F}}\left(\tilde{V}\right)$ denotes the nonmembership degree.

**Example 3 .**

Let $G=\left(\tilde{V},\text{Ê}\right)$ be a SVNG, where $\tilde{V}$ is the collection of vertices and Ê is the collection of edges

**Figure 3:**
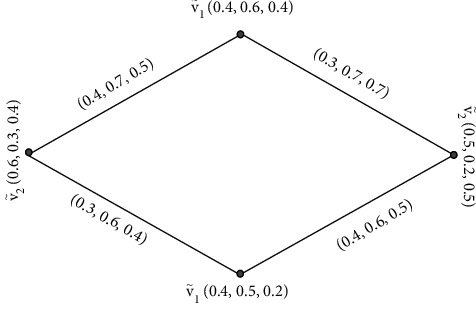
Degrees of single-valued neutrosophic graph.

Figure 3 is an NG which is explained below.

This graph contains four vertices  *v*_1_,  *v*_2_, *v*_3_,  and *v*_4_, and the values between their vertices is called edges. Furthermore, by Definition 4, we find the degrees of its vertices of Figure 3 which is given below.

Degree of vertices of Figure 3 is

(1) $$\begin{array}{c} \deg\left(\;{\tilde{V}}_{1}\right)=\left(0.7,1.4,1.2\right),\\ \deg\left(\;{\tilde{V}}_{2}\right)=\left(0.7,1.3,1.2\right),\\ \deg\left(\;{\tilde{V}}_{3}\right)=\left(0.7,1.2,0.9\right),\\ \deg\left(\;{\tilde{V}}_{4}\right)=\left(0.7,1.3,0.9\right). \end{array}$$

**Definition 5 (see [27]).**

The SVN-weighted aggregation (SVNWA) operator is denoted and defined by ${𝒩}_{i}=\text{SVNWA}\left({𝒩}_{i1},{𝒩}_{i2},\ldots ,{𝒩}_{in}\right)=\left(1-\prod_{j=1}^{n}{\left(1-T_{ij}\right)}^{w_{j}},{\left(\prod_{j=1}^{n}L_{ij}\right)}^{w_{j}},{\left(\prod_{j=1}^{n}{\underline{F}}_{ij}\right)}^{w_{j}}\right)$,  *i*=1,2,…, *n* , where ${\ddot{W}}_{j}\left(1,2,\ldots ,n\right)$ represents the weight vector.

**Definition 6 (see [27]).**

The SVN-weighted geometric (SVNWG) operator is denoted and defined by

(2) $$\begin{array}{c} N_{i}=\text{SVNWG}\left(N_{i1},N_{i2},\ldots ,N_{in}\right)=\left({\left(\prod_{j=1}^{n}{\ddot{W}}_{ij}\right)}^{{\ddot{W}}_{j}},1-\prod_{j=1}^{n}{\left(1-{\ddot{W}}_{ij}\right)}^{{\ddot{W}}_{j}},1-\prod_{j=1}^{n}{\left(1-{\dot{F}}_{ij}\right)}^{{\ddot{W}}_{j}}\right),\;i=1,2,\ldots ,n, \end{array}$$

where ${\ddot{W}}_{j}\left(1,2,\ldots ,n\right)$ represent the weight vector.

**Definition 7 (see [28]).**

The single-valued neutrosophic Hamming distance between two SVNSs (*𝒩*_*i*_, *𝒩*_*j*_) is defined by

(3) $$\begin{array}{c} \underline{D}\left(N_{i},N_{j}\right)=\frac{1}{3n}\overset{n}{\underset{j=1}{\sum }}\left|{\underline{T}}_{N_{i}}\left({\underline{Z}}_{i}\right)-{\underline{T}}_{N_{j}}\left({\underline{Z}}_{j}\right)\right|+\left|{\underset{⌢}{I}}_{N_{i}}\left({\underline{Z}}_{i}\right)-{\underset{⌢}{I}}_{N_{j}}\left({\underline{Z}}_{j}\right)\right|+\left|{\dot{F}}_{N_{i}}\left({\underline{Z}}_{i}\right)-{\dot{F}}_{N_{j}}\left({\underline{Z}}_{j}\right)\right|. \end{array}$$

**Definition 8 (see [27]).**

The score function in a SVNS is denoted and defined by $\;\underset{˙}{S}\left({𝒩}_{i}\right)=\left(\underline{T}+1-\underset{⌢}{I}+1-\dot{F}\right)/3\;$, where $\left(\underline{T},\underset{⌢}{I},\dot{F}\right)$ represents the membership, indeterminacy, and nonmembership grades, respectively.

## 3. Edge Irregular and Highly Edge Irregular SVNG

We propose the definitions of edge irregular and highly edge irregular SVNG in this section.

**Definition 9 .**

A connected graph $\overset{\cdot }{G}=\left(\tilde{V},\text{Ê}\right)$ is called the edge irregular SVNG (EISVNG) if at least single edge is neighboring to the edges with different degrees.

**Definition 10 .**

A connected graph $\overset{\cdot }{G}=\left(\tilde{V},\text{Ê}\right)$ is called an edge totally irregular SVNG (ETISVNG) if at least single edge is neighboring the edges with different total degrees.

**Definition 11 .**

A connected graph $\overset{\cdot }{G}=\left(\tilde{V},\text{Ê}\right)$ is called highly edge irregular SVNG (HEISVNG) if each edge is neighboring to the edges with different degrees.

**Example 4 .**

Let $\overset{\cdot }{G}=\left(\tilde{V},\text{Ê}\right)$ be a SVNG, where Ê is the collection of edges and $\tilde{V}$ is the collection of vertices.

**Figure 4:**
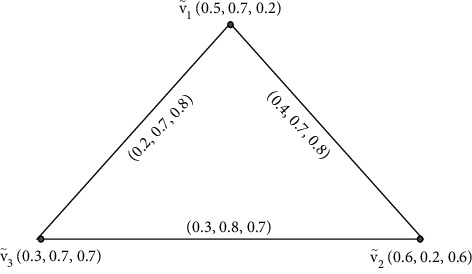
Edge irregular single-valued neutrosophic graph.

Figure 4 contains four vertices  *v*_1_,  *v*_2_, *v*_3_,  and *v*_4_, and the values between their vertices are called edges. Furthermore, by Definition 4, we find the degrees of its vertices of Figure 4.

Here, ${ď}_{\overset{\cdot }{G}}\left({\tilde{V}}_{1}\right)=\left(0.6,1.4,1.6\right)$, ${ď}_{\overset{\cdot }{G}}\left({\tilde{V}}_{2}\right)=\left(0.7,1.5,1.5\right)$, and ${ď}_{\overset{\cdot }{G}}\left({\tilde{V}}_{3}\right)=\left(0.5,1.5,1.5\right)$. Degrees of edges are

(4) $$\begin{array}{c} {ď}_{\overset{\cdot }{G}}\left({\tilde{V}}_{1}{\tilde{V}}_{2}\right)=\left(0.6+0.7-2\times 0.4,1.4+1.5-2\times 0.7,1.6+1.5-2\times 0.8\right)=\left(0.5,1.5,\;1.5\right),\\ {ď}_{\overset{\cdot }{G}}\left({\tilde{V}}_{2}{\tilde{V}}_{3}\right)=\left(0.7+0.5-2\times 0.3,1.5+1.5-2\times 0.8,1.5+1.5-2\times 0.7\right)=\left(0.6,1.4,1.6\right),\\ {ď}_{\overset{\cdot }{G}}\left({\tilde{V}}_{3}{\tilde{V}}_{1}\right)=\left(0.6+0.5-2\times 0.2,1.4+1.5-2\times 0.8,1.6+1.5-2\times 0.8\right)=\left(0.7,1.3,1.5\right). \end{array}$$

We observe that every edge is neighboring to the edges with different degrees. Consequently, $\overset{\cdot }{G}$ is HEISVNG and also EISVNG.

**Definition 12 .**

A connected graph $\overset{\cdot }{G}=\left(\tilde{V},\text{Ê}\right)$ is called HETISVNG if each edge is neighboring to the edges with different total degrees.

We also propose the following theorems as statements that have been proven to be true.

**Theorem 1 .**

If $\overset{\cdot }{G}=\left(\tilde{V},\text{Ê}\right)$ is a connected HEISVNG, then $\overset{\cdot }{G}$ is an EISVNG.

**Proof**

Let us assume that $\overset{\cdot }{G}$ is a connected HEISVNG; then, each edge in $\overset{\cdot }{G}$ neighbors the edges with different degrees; consequently, there exist at least single edge that is neighboring the edge with distinct degrees. Hence, $\overset{\cdot }{G}$ is an EISVNG.

**Theorem 2 .**

If $\overset{\cdot }{G}=\left(\tilde{V},\text{Ê}\right)$ is a connected HETISVNG, then $\overset{\cdot }{G}$ is an ETISVNG.

**Proof**

It follows Theorem 1, thus omitted.

**Remark 1 .**

A HEISVNG may not be a HETISVNG.

**Example 5 .**

This example supports Remark 1.

Let $\overset{\cdot }{G}=\left(\tilde{V},\text{Ê}\right)$ be a SVNG.

**Figure 5:**
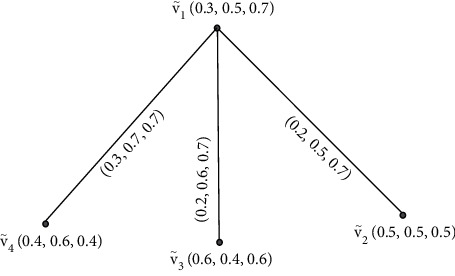
Highly edge irregular singular-valued neutrosophic graph.

Figure 5 contains four vertices *v*_1_,  *v*_2_, *v*_3_,  and *v*_4_, and the values between their vertices is called edges. Furthermore, by Definition 4, we find the degrees of its vertices are given as below.

Here, ${ď}_{\overset{\cdot }{G}}\left({\tilde{V}}_{1}\right)=\left(0.7,1.8,1.6\right)$, ${ď}_{\overset{\cdot }{G}}\left({\tilde{V}}_{2}\right)=\left(0.2,0.5,0.7\right)$, ${ď}_{\overset{\cdot }{G}}\left({\tilde{V}}_{3}\right)=\left(0.2,0.6,0.7\right)$, and ${ď}_{\overset{\cdot }{G}}\left({\tilde{V}}_{4}\right)=\left(0.3,0.7,0.7\right)$. Degrees of edges are ${ď}_{\overset{\cdot }{G}}\left({\tilde{V}}_{1},{\tilde{V}}_{2}\right)=\left(0.7+0.2-2\times 0.2,1.8+0.5-2\times 0.5,1.6+0.7-2\times 0.7\right)=\left(0.6,1.3,0.9\right)$, ${ď}_{\overset{\cdot }{G}}\left({\tilde{V}}_{2},{\tilde{V}}_{3}\right)=\left(0.7+0.2-2\times 0.2,1.8+0.6-2\times 0.6,1.6+0.7-2\times 0.7\right)=\left(0.5,1.2,0.9\right)$, and ${ď}_{\overset{\cdot }{G}}\left({\tilde{V}}_{1},{\tilde{V}}_{4}\right)=\left(0.7+0.3-2\times 0.3,1.8+0.7-2\times 0.7,1.6+0.7-2\times 0.7\right)$ and $t{ď}_{\overset{\cdot }{G}}\left({\tilde{V}}_{1},{\tilde{V}}_{2}\right)=\left(0.7+0.2-0.2,1.8+0.5-0.5,1.6+0.7-0.7\right)=\left(0.7,1.8,1.6\right),$$t{ď}_{\overset{\cdot }{G}}\left({\tilde{V}}_{1},{\tilde{V}}_{3}\right)=\left(0.7+0.2-0.2,1.8+0.6-0.6,1.6+0.7-0.7\right)=\left(0.7,1.8,1.6\right)$, and $t{ď}_{\overset{\cdot }{G}}\left({\tilde{V}}_{1},{\tilde{V}}_{4}\right)=\left(0.7+0.3-0.3,1.8+0.7-0.7,1.6+0.7-0.7\right)=\left(0.7,1.8,1.6\right).$ Clearly, we note that $\overset{\cdot }{G}$ is HEISVNG, but $\overset{\cdot }{G}$ is not HETISVNG. Therefore, all edges are with the same total degrees.

**Remark 2 .**

HETISVNG might not be an HEISVNG.

**Example 6 .**

This example supports Remark 2.

Let $\overset{\cdot }{G}=\left(\tilde{V},\text{Ê}\right)$ be an SVNG.

**Figure 6:**
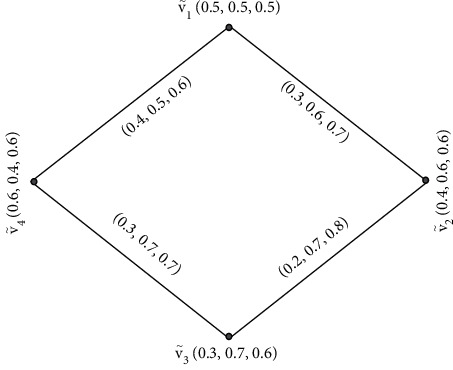
Highly edge totally irregular single-valued neutrosophic graph.

Figure 6 contains four vertices *v*_1_, *v*_2_, *v*_3_,  and *v*_4_, and the values between their vertices are called edges. Furthermore, by Definition 4, we find the degrees of its vertices of Figure 6.

Here, ${ď}_{\overset{\cdot }{G}}\left({\tilde{V}}_{1}\right)=\left(0.7,1.1,1.3\right)$, ${ď}_{\overset{\cdot }{G}}\left({\tilde{V}}_{2}\right)=\left(0.5,1.3,1.5\right)$, ${ď}_{\overset{\cdot }{G}}\left({\tilde{V}}_{3}\right)=\left(0.5,1.4,1.5\right)$, and ${ď}_{\overset{\cdot }{G}}\left({\tilde{V}}_{4}\right)=\left(0.7,1.2,1.3\right)$. Degrees of edges are ${ď}_{\overset{\cdot }{G}}\left({\tilde{V}}_{1},{\tilde{V}}_{2}\right)=\left(0.7+0.5-2\times 0.2,1.8+0.5-2\times 0.5,1.6+0.7-2\times 0.7\right)=\left(0.8,1.3,0.9\right)$, ${ď}_{\overset{\cdot }{G}}\left({\tilde{V}}_{1},{\tilde{V}}_{3}\right)=\left(0.7+0.2-2\times 0.2,\;1.8+0.6-2\times 0.6,\;1.6+0.7-2\times 0.7\right)=\left(0.5,\;1.2,\;0.9\right)$, and ${ď}_{\overset{\cdot }{G}}\left({\tilde{V}}_{1},{\tilde{V}}_{4}\right)=\left(0.7+0.3-2\times 0.3,1.8+0.7-2\times 0.7,1.6+0.7-2\times 0.7\right)=\left(0.4,1.1,0.9\right)$ and $t{ď}_{\overset{\cdot }{G}}\left({\tilde{V}}_{1},\;{\tilde{V}}_{2}\right)=\left(0.7+0.2-0.2,1.8+0.5-0.5,1.6+0.7-0.7\right)=\left(0.7,1.8,1.6\right),$$t{ď}_{\overset{\cdot }{G}}\left({\tilde{V}}_{1},\;{\tilde{V}}_{3}\right)=\left(0.7+0.2-0.2,1.8+0.6-0.6,1.6+0.7-0.7\right)=\left(0.7,1.8,1.6\right)$, and $t{ď}_{\overset{\cdot }{G}}\left({\tilde{V}}_{1},{\tilde{V}}_{4}\right)=\left(0.7+0.3-0.3,1.8+0.7-0.7,1.6+0.7-0.7\right)=\left(0.7,1.8,1.6\right).$

**Theorem 3 .**

If a connected SVNG $\overset{\cdot }{G}=\left(\tilde{V},\text{Ê}\right)$ is HISVNG and Ê is constant function, then $\overset{\cdot }{G}$ is HETISVNG.

**Proof**

Suppose that Ê is constant function. Assume that $e_{1}\left(U^{\prime },\tilde{V}\right)=\varsigma_{1}$, $e_{2}\left(U^{\prime },\tilde{V}\right)=\varsigma_{2}$, and $e_{3}\left(U^{\prime },\tilde{V}\right)=\varsigma_{3}$; for all $e_{i}\left(U^{\prime },\tilde{V}\right)\in \text{Ê}$, *ς*_1_, *ς*_2_, and *ς*_3_ are constants. Now, consider $\overset{\cdot }{G}$ is EHISVNG. Then, every edge is neighboring to the edges; it has distinct degrees. Suppose $\left(U^{\prime },\tilde{V}\right)$ be an edge that is neighboring to the edges (*U*′, *ω*) and (*U*′, *X*), and these edges that are incident at the vertex *U*′ and $\left(\tilde{V},y\right)$ are the edge incident with the vertex $\tilde{V}$ Then, ${ď}_{1\overset{\cdot }{G}}\left(U^{\prime },\omega \right)\neq {ď}_{1\overset{\cdot }{G}}\left(U^{\prime },x\right)\neq {ď}_{1\overset{\cdot }{G}}\left(\tilde{V},y\right)$, ${ď}_{2\overset{\cdot }{G}}\left(U^{\prime },\omega \right)\neq {ď}_{2\overset{\cdot }{G}}\left(U^{\prime },x\right)\neq {ď}_{2\overset{\cdot }{G}}\left(\tilde{V},y\right)$, and ${ď}_{3\overset{\cdot }{G}}\left(U^{\prime },\omega \right)\neq {ď}_{3\overset{\cdot }{G}}\left(U^{\prime },x\right)\neq {ď}_{3\overset{\cdot }{G}}\left(\tilde{V},y\right)$, where (*U*′, *ω*), (*U*′, *x*), and $\left(\tilde{V},y\right)$ are neighboring to the vertex $\left(U^{\prime },\tilde{V}\right)\in \text{Ê}.$ Next, ${ď}_{1\overset{\cdot }{G}}\left(U^{\prime },\omega \right)\neq {ď}_{1\overset{\cdot }{G}}\left(U^{\prime },x\right)\neq {ď}_{1\overset{\cdot }{G}}\left(\tilde{V},Y\right)\Rightarrow {ď}_{1\overset{\cdot }{G}}\left(U^{\prime },\omega \right)\varsigma_{1}\neq {ď}_{1\overset{\cdot }{G}}\left(U^{\prime },x\right)+\varsigma_{1}\neq {ď}_{1\overset{\cdot }{G}}\left(\tilde{V},Y\right)+\varsigma_{1}$⇒${ď}_{1\overset{\cdot }{G}}\left(U^{\prime },\omega \right)+\;e_{1}\left(U^{\prime },\omega \right)\neq {ď}_{1\overset{\cdot }{G}}\left(U^{\prime },x\right)+\;e_{1}\left(U^{\prime },x\right)\neq {ď}_{1\overset{\cdot }{G}}\left(\tilde{V},y\right)+\;e_{1}\left(\tilde{V},y\right)\Rightarrow t{ď}_{1\overset{\cdot }{G}}\left(U^{\prime },\omega \right)\neq t{ď}_{1\overset{\cdot }{G}}\left(U^{\prime },x\right)\neq t{ď}_{1\overset{\cdot }{G}}\left(\tilde{V},y\right).$ Again, ${ď}_{2\overset{\cdot }{G}}\left(U^{\prime },\omega \right)\neq {ď}_{2\overset{\cdot }{G}}\left(U^{\prime },x\right)\neq {ď}_{2\overset{\cdot }{G}}\left(\tilde{V},y\right)\Rightarrow {ď}_{2\overset{\cdot }{G}}\left(U^{\prime },\omega \right)+\varsigma_{2}\neq {ď}_{2\overset{\cdot }{G}}\left(U^{\prime },x\right)+\varsigma_{2}\neq {ď}_{2\overset{\cdot }{G}}\left(\tilde{V},y\right)+\varsigma_{2}\Rightarrow$${ď}_{2\overset{\cdot }{G}}\left(U^{\prime },\omega \right)+\;e_{2}\left(U^{\prime },\omega \right)\neq {ď}_{2\overset{\cdot }{G}}\left(U^{\prime },x\right)+\;e_{2}\left(U^{\prime },x\right)\neq {ď}_{2\overset{\cdot }{G}}\left(\tilde{V},y\right)+\;e_{2}\left(\tilde{V},y\right)\Rightarrow t{ď}_{2\overset{\cdot }{G}}\left(U^{\prime },\omega \right)\neq t{ď}_{2\overset{\cdot }{G}}\left(U^{\prime },x\right)\neq t{ď}_{2\overset{\cdot }{G}}\left(\tilde{V},y\right)$ and ${ď}_{3\overset{\cdot }{G}}\left(U^{\prime },\omega \right)\neq {ď}_{3\overset{\cdot }{G}}\left(U^{\prime },x\right)\neq {ď}_{3\overset{\cdot }{G}}\left(\tilde{V},y\right)\Rightarrow {ď}_{3\overset{\cdot }{G}}\left(U^{\prime },\omega \right)\neq {ď}_{3\overset{\cdot }{G}}\left(U^{\prime },x\right)\neq {ď}_{3\overset{\cdot }{G}}\left(\tilde{V},y\right)+\varsigma_{3}$⇒${ď}_{3\overset{\cdot }{G}}\left(U^{\prime },\omega \right)+\;e_{3}\left(U^{\prime },\omega \right)\neq {ď}_{3\overset{\cdot }{G}}\left(U^{\prime },x\right)+\;e_{3}\left(U^{\prime },x\right)\neq {ď}_{3\overset{\cdot }{G}}\left(\tilde{V},y\right)+\;e_{3}\left(\tilde{V},y\right)\Rightarrow t{ď}_{3\overset{\cdot }{G}}\left(U^{\prime },\omega \right)\neq t{ď}_{3\overset{\cdot }{G}}\left(U^{\prime },x\right)\neq t{ď}_{3\overset{\cdot }{G}}\left(\tilde{V},y\right)$. Hence, $\overset{\cdot }{G}$ is HETISVNG.

**Theorem 4 .**

If a connected SVNG $\overset{\cdot }{G}=\left(\tilde{V},\text{Ê}\right)$ is EISVNG and Ê is constant function, then $\overset{\cdot }{G}$ is ETISVNG.

**Proof**

It follows the proof Theorem 3, and thus, it is omitted.

**Theorem 5 .**

If a connected SVNG $\overset{\cdot }{G}=\left(\tilde{V},\text{Ê}\right)$ is ETISVNG and Ê is constant function, then $\overset{\cdot }{G}$ is EISVNG.

**Proof**

It follows the proof of Theorem 3, and thus, it is omitted.

**Remark 3 .**

If a connected SVNG $\overset{\cdot }{G}=\left(\tilde{V},\text{Ê}\right)$ is both HEISVNG and HETISVNG. Then, Ê may not be considered a constant function.

**Example 7 .**

The following example supports Remark 3.

Let $\overset{\cdot }{G}=\left(\tilde{V},\text{Ê}\right)$ be a SVNG.

**Figure 7:**
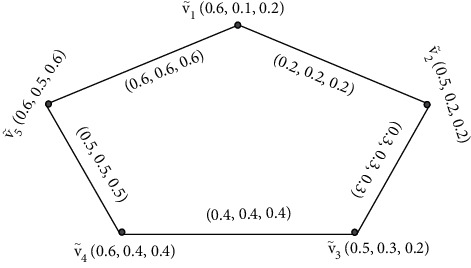
Highly edge and highly edge totally irregular single-valued neutrosophic graph.

Figure 7 contains four vertices  *v*_1_,  *v*_2_, *v*_3_,  and *v*_4_, and the values between their vertices are called edges. Furthermore, by Definition 4, we find the degrees of its vertices of Figure 7.

Here, ${ď}_{\overset{\cdot }{G}}\left({\tilde{V}}_{1}\right)=\left(0.8,0.8,0.8\right)$, ${ď}_{\overset{\cdot }{G}}\left({\tilde{V}}_{2}\right)=\left(0.5,0.5,0.5\right)$, ${ď}_{\overset{\cdot }{G}}\left({\tilde{V}}_{3}\right)=\left(0.7,0.7,0.7\right)$, ${ď}_{\overset{\cdot }{G}}\left({\tilde{V}}_{4}\right)=\left(0.9,0.9,0.9\right)$, and ${ď}_{\overset{\cdot }{G}}\left({\tilde{V}}_{5}\right)=\left(1.1,1.1,1.1\right)$ Degrees of edges are ${ď}_{\overset{\cdot }{G}}\left({\tilde{V}}_{1}{\tilde{V}}_{2}\right)=\left(0.7+0.5-2\times 0.2,1.8+0.5-2\times 0.5,1.6+0.7-2\times 0.7\right)=\left(0.8,1.3,0.9\right)$, ${ď}_{\overset{\cdot }{G}}\left({\tilde{V}}_{1}{\tilde{V}}_{3}\right)=\left(0.7+0.2-2\times 0.2,1.8+0.6-2\times 0.6,1.6+0.7-2\times 0.7\right)=\left(0.5,1.2,\;0.9\right)$, and ${ď}_{\overset{\cdot }{G}}\left({\tilde{V}}_{1}{\tilde{V}}_{4}\right)=\left(0.7+0.3-2\times 0.3,1.8+0.7-2\times 0.7,1.6+0.7-2\times 0.7\right)=\left(0.4,1.1,0.9\right)$ and $t{ď}_{\overset{\cdot }{G}}\left({\tilde{V}}_{1},{\tilde{V}}_{2}\right)=\left(0.7+0.2-0.2,1.8+0.5-0.5,1.6+0.7-0.7\right)=\left(0.7,1.8,1.6\right)$, $t{ď}_{\overset{\cdot }{G}}\left({\tilde{V}}_{1},{\tilde{V}}_{3}\right)=\left(0.7+0.2-0.2,1.8+0.6-0.6,1.6+0.7-0.7\right)=\left(0.7,1.8,1.6\right)$, and $t{ď}_{\overset{\cdot }{G}}\left({\tilde{V}}_{1},{\tilde{V}}_{4}\right)=\left(0.7+0.3-0.3,1.8+0.7-0.7,1.6+0.7-0.7\right)=\left(0.7,1.8,1.6\right)$.

**Theorem 6 .**

If a connected SVNG $\overset{\cdot }{G}=\left(\tilde{V},\text{Ê}\right)$ is EISVNG and Ê is constant function, then $\overset{\cdot }{G}$ is an ISVNG.

**Proof**

Suppose that Ê is constant function. Assume that $e_{1}\left(U^{\prime },\tilde{V}\right)=\varsigma_{1}$, $e_{2}\left(U^{\prime },\tilde{V}\right)=\varsigma_{2}$, and $e_{3}\left(U^{\prime },\tilde{V}\right)=\varsigma_{3}$, for all $e_{i}\left(U^{\prime },\tilde{V}\right)\in \text{Ê}$,  *ς*_1_, *ς*_2_, and  *ς*_3_ are constants. Now, consider $\overset{\cdot }{G}$ is EHISVNG. Then, every edge neighbors the edges with distinct degrees. Suppose $\left(U^{\prime },\tilde{V}\right)$ be an edge such that it is neighboring the edges (*U*′, *ω*), (*U*′, *x*), and these edges are incident at the vertex *U*′ and $\left(\tilde{V},y\right)$ is the edge incident to the vertex *U*′. Then, ${ď}_{1\overset{\cdot }{G}}\left(U^{\prime },\omega \right)\neq {ď}_{1\overset{\cdot }{G}}\left(U^{\prime },x\right)\neq {ď}_{1\overset{\cdot }{G}}\left(\tilde{V},y\right)$, ${ď}_{2\overset{\cdot }{G}}\left(U^{\prime },\omega \right)\neq {ď}_{2\overset{\cdot }{G}}\left(U^{\prime },x\right)\neq {ď}_{2\overset{\cdot }{G}}\left(\tilde{V},y\right)$, and ${ď}_{3\overset{\cdot }{G}}\left(U^{\prime },\omega \right)\neq {ď}_{3\overset{\cdot }{G}}\left(U^{\prime },x\right)\neq {ď}_{3\overset{\cdot }{G}}\left(\tilde{V},y\right)$, where (*U*′, *ω*), (*U*′, *x*), and $\left(\tilde{V},y\right)$ are neighboring to the vertex $\left(U^{\prime },\tilde{V}\right)\in \text{Ê}$. Now, $\begin{array}{c} {ď}_{1\overset{\cdot }{G}}\left(U^{\prime },\omega \right)\neq {ď}_{1\overset{\cdot }{G}}\left(U^{\prime },x\right)\neq {ď}_{1\overset{\cdot }{G}}\left(\tilde{V},y\right)\Rightarrow {ď}_{1\overset{\cdot }{G}}\left(U^{\prime }\right)+\;{ď}_{1\overset{\cdot }{G}}\left(\omega \right)-2\;e_{1}\left(U^{\prime },\tilde{V}\right)\neq {ď}_{1\overset{\cdot }{G}}\left(U^{\prime }\right)+{ď}_{1\overset{\cdot }{G}}\left(x\right)-2\;e_{1}\left(U^{\prime },x\right)\neq {ď}_{1\overset{\cdot }{G}}\left(\tilde{V}\right)+{ď}_{1\overset{\cdot }{G}}\left(y\right)-2\;e_{1}\left(\tilde{V},y\right)\Rightarrow {ď}_{1\overset{\cdot }{G}}\left(U^{\prime }\right)\\ +\;{ď}_{1\overset{\cdot }{G}}\left(\omega \right)-2\varsigma_{1}\neq {ď}_{1\overset{\cdot }{G}}\left(U^{\prime }\right)+{ď}_{1\overset{\cdot }{G}}\left(x\right)-2\varsigma_{1}\neq {ď}_{1\overset{\cdot }{G}}\left(\tilde{V}\right)+{ď}_{1\overset{\cdot }{G}}\left(y\right)-2\varsigma_{1}\Rightarrow {ď}_{1\overset{\cdot }{G}}\left(U^{\prime }\right)+{ď}_{1\overset{\cdot }{G}}\left(\omega \right)\neq {ď}_{1\overset{\cdot }{G}}\left(U^{\prime }\right)+{ď}_{1\overset{\cdot }{G}}\left(x\right)\neq {ď}_{1\overset{\cdot }{G}}\left(\tilde{V}\right)+{ď}_{1\overset{\cdot }{G}}\left(y\right)\Rightarrow {ď}_{1\overset{\cdot }{G}}\left(\omega \right)\neq {ď}_{1\overset{\cdot }{G}}\left(x\right) \end{array}$. Again, $\begin{array}{c} {ď}_{2\overset{\cdot }{G}}\left(U^{\prime },\omega \right)\neq {ď}_{2\overset{\cdot }{G}}\left(U^{\prime },x\right)\neq {ď}_{2\overset{\cdot }{G}}\left(\tilde{V},y\right)\Rightarrow {ď}_{2\overset{\cdot }{G}}\left(U^{\prime }\right)+\;{ď}_{2\overset{\cdot }{G}}\left(\omega \right)-2\;e_{2}\left(U^{\prime },\tilde{V}\right)\neq {ď}_{2\overset{\cdot }{G}}\left(U^{\prime }\right)+{ď}_{2\overset{\cdot }{G}}\left(x\right)-2\;e_{2}\left(U^{\prime },x\right)\neq {ď}_{2\overset{\cdot }{G}}\left(\tilde{V}\right)+{ď}_{2\overset{\cdot }{G}}\left(y\right)-2\;e_{2}\left(\tilde{V},y\right)\Rightarrow {ď}_{2\overset{\cdot }{G}}\left(U^{\prime }\right)\\ +\;{ď}_{2\overset{\cdot }{G}}\left(\omega \right)-2\varsigma_{2}\neq {ď}_{2\overset{\cdot }{G}}\left(U^{\prime }\right)+{ď}_{2\overset{\cdot }{G}}\left(x\right)-2\varsigma_{2}\neq {ď}_{2\overset{\cdot }{G}}\left(\tilde{V}\right)+{ď}_{2\overset{\cdot }{G}}\left(y\right)-2\varsigma_{2}\Rightarrow {ď}_{2\overset{\cdot }{G}}\left(U^{\prime }\right)+{ď}_{2\overset{\cdot }{G}}\left(\omega \right)\neq {ď}_{2\overset{\cdot }{G}}\left(U^{\prime }\right)+{ď}_{2\overset{\cdot }{G}}\left(x\right)\neq {ď}_{2\overset{\cdot }{G}}\left(\tilde{V}\right)+{ď}_{2\overset{\cdot }{G}}\left(y\right)\Rightarrow {ď}_{2\overset{\cdot }{G}}\left(\omega \right)\neq {ď}_{2\overset{\cdot }{G}}\left(x\right) \end{array}$ and $\begin{array}{c} {ď}_{3\overset{\cdot }{G}}\left(U^{\prime },\omega \right)\neq {ď}_{3\overset{\cdot }{G}}\left(U^{\prime },x\right)\neq {ď}_{3\overset{\cdot }{G}}\left(\tilde{V},y\right)\Rightarrow {ď}_{3\overset{\cdot }{G}}\left(U^{\prime }\right)+\;{ď}_{3\overset{\cdot }{G}}\left(\omega \right)-2\;e_{3}\left(U^{\prime },\tilde{V}\right)\neq {ď}_{3\overset{\cdot }{G}}\left(U^{\prime }\right)+{ď}_{3\overset{\cdot }{G}}\left(x\right)-2\;e_{3}\left(U^{\prime },x\right)\neq {ď}_{3\overset{\cdot }{G}}\left(\tilde{V}\right)+{ď}_{3\overset{\cdot }{G}}\left(y\right)-2\;e_{3}\left(\tilde{V},y\right)\Rightarrow {ď}_{3\overset{\cdot }{G}}\left(U^{\prime }\right)\\ +\;{ď}_{3\overset{\cdot }{G}}\left(\omega \right)-2\varsigma_{3}\neq {ď}_{3\overset{\cdot }{G}}\left(U^{\prime }\right)+{ď}_{3\overset{\cdot }{G}}\left(x\right)-2\varsigma_{3}\neq {ď}_{3\overset{\cdot }{G}}\left(\tilde{V}\right)+{ď}_{3\overset{\cdot }{G}}\left(y\right)-2\varsigma_{3}\Rightarrow {ď}_{3\overset{\cdot }{G}}\left(U^{\prime }\right)+{ď}_{3\overset{\cdot }{G}}\left(\omega \right)\neq {ď}_{3\overset{\cdot }{G}}\left(U^{\prime }\right)+{ď}_{3\overset{\cdot }{G}}\left(x\right)\neq {ď}_{3\overset{\cdot }{G}}\left(\tilde{V}\right)+{ď}_{3\overset{\cdot }{G}}\left(y\right)\Rightarrow {ď}_{3\overset{\cdot }{G}}\left(\omega \right)\neq {ď}_{3\overset{\cdot }{G}}\left(x\right) \end{array}$.

Consequently, there is a vertex *U*′ neighboring the vertices *ω* and *x* with different degrees. Thus, $\overset{\cdot }{G}$ is an ISVNG. In Section 4, we present several definitions and examples to explain the degree of edge irregularity.

## 4. Strongly Edge Irregular and Strongly Edge Totally Irregular SVNG

**Definition 13 .**

A connected graph $\overset{\cdot }{G}=\left(\tilde{V},\text{Ê}\right)$ is known as strongly EISVNG if each pair of edges has different degrees.

**Definition 14 .**

A connected graph $\overset{\cdot }{G}=\left(\tilde{V},\text{Ê}\right)$ is called strongly ETISVNG if each pair of edges has different total degrees.

**Example 8 .**

The following example supports Remark 3. Let $\overset{\cdot }{G}=\left(\tilde{V},\text{Ê}\right)$ be a SVNG.

**Figure 8:**
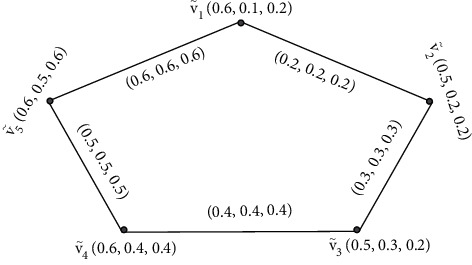
Strongly edge and strongly edge totally irregular single-valued neutrosophic graph.

Figure 8 contains five vertices  *v*_1_,  *v*_2_, *v*_4_, *v*_4_,  and *v*_5_, and the values between their vertices are called edges. Furthermore, by Definition 4, we find the degrees of its vertices of Figure 8.

Here, ${ď}_{\overset{\cdot }{G}}\left({\tilde{V}}_{1}\right)=\left(0.8,0.8,0.8\right)$, ${ď}_{\overset{\cdot }{G}}\left({\tilde{V}}_{2}\right)=\left(0.5,0.5,0.5\right)$, ${ď}_{\overset{\cdot }{G}}\left({\tilde{V}}_{3}\right)=\left(0.7,0.7,0.7\right)$, ${ď}_{\overset{\cdot }{G}}\left({\tilde{V}}_{5}\right)=\left(0.9,0.9,0.9\right)$, and ${ď}_{\overset{\cdot }{G}}\left({\tilde{V}}_{5}\right)=\left(1.1,1.1,1.1\right)$. Degrees of edges are ${ď}_{\overset{\cdot }{G}}\left({\tilde{V}}_{1},{\tilde{V}}_{2}\right)=\left(0.7+0.5-2\times 0.2,1.8+0.5-2\times 0.5,1.6+0.7-2\times 0.7\right)=\left(0.8,1.3,0.9\right)$, ${ď}_{\overset{\cdot }{G}}\left({\tilde{V}}_{1},{\tilde{V}}_{3}\right)=\left(0.7+0.2-2\times 0.2,1.8+0.6-2\times 0.6,1.6+0.7-2\times 0.7\right)=\left(0.5,1.2,0.9\right)$, and ${ď}_{\overset{\cdot }{G}}\left({\tilde{V}}_{1},{\tilde{V}}_{4}\right)=\left(0.7+0.3-2\times 0.3,1.8+0.7-2\times 0.7,1.6+0.7-2\times 0.7\right)=\left(0.4,1.1,0.9\right)$ and $t{ď}_{\overset{\cdot }{G}}\left({\tilde{V}}_{1},{\tilde{V}}_{2}\right)=\left(0.7+0.2-0.2,1.8+0.5-0.5,1.6+0.7-0.7\right)=\left(0.7,1.8,1.6\right)$, $t{ď}_{\overset{\cdot }{G}}\left({\tilde{V}}_{1},{\tilde{V}}_{3}\right)=\left(0.7+0.2-0.2,1.8+0.6-0.6,1.6+0.7-0.7\right)=\left(0.7,1.8,1.6\right)$, and $t{ď}_{\overset{\cdot }{G}}\left({\tilde{V}}_{1},\;{\tilde{V}}_{4}\right)=\left(0.7+0.3-0.3,1.8+0.7-0.7,1.6+0.7-0.7\right)=\left(0.7,1.8,1.6\right)$.

**Theorem 7 .**

If $\overset{\cdot }{G}=\left(\tilde{V},\text{Ê}\right)$ is a strongly connected EISVNG, then $\overset{\cdot }{G}$ is an HEISVNG.

**Proof**

Let us assume that $\overset{\cdot }{G}$ is a connected strongly EISVNG; then, all pairs of edges in $\overset{\cdot }{G}$ have distinct degrees; therefore, every edge neighbors the edge with a distinct degree. Hence, $\overset{\cdot }{G}$ is an HEISVNG.

**Theorem 8 .**

If a connected SVNG $\overset{\cdot }{G}=\left(\tilde{V},\text{Ê}\right)$ is strongly EISVNG and Ê is constant function, then $\overset{\cdot }{G}$ is strongly ETISVNG.

**Proof**

Suppose that Ê is constant function. Assume that $e_{1}\left(U^{\prime },\tilde{V}\right)=\varsigma_{1}$, $e_{2}\left(U^{\prime },\tilde{V}\right)=\varsigma_{2}$, and $e_{3}\left(U^{\prime },\tilde{V}\right)=\varsigma_{3}$; for all $e_{i}\left(U^{\prime },\tilde{V}\right)\in \text{Ê}$,  *ς*_1_, *ς*_2_, and  *ς*_3_ are constants. Now, assume that $\overset{\cdot }{G}$ is strongly EISVNG. Then, every pair of edge is neighboring to the edges with distinct degrees. Suppose $\left(U^{\prime },\tilde{V}\right)$ and (*x*, *y*) be some pair of edge in Ê. Now, ${ď}_{1\overset{\cdot }{G}}\left(U^{\prime },\tilde{V}\right)\neq {ď}_{1\overset{\cdot }{G}}\left(x,y\right)\Rightarrow {ď}_{1\overset{\cdot }{G}}\left(U^{\prime },\tilde{V}\right)+\varsigma_{1}\neq {ď}_{1\overset{\cdot }{G}}\left(x,y\right)+\varsigma_{1}$$\Rightarrow {ď}_{1\overset{\cdot }{G}}\left(U^{\prime },\tilde{V}\right)+\;e_{1}\left(U^{\prime },\tilde{V}\right)\neq {ď}_{1\overset{\cdot }{G}}\left(x,y\right)+\;e_{1}\left(x,y\right)\Rightarrow t{ď}_{1\overset{\cdot }{G}}\left(U^{\prime },\tilde{V}\right)\neq t{ď}_{1\overset{\cdot }{G}}\left(x,y\right)$ for any pair of $\left(U^{\prime },\tilde{V}\right)$ and (*x*, *y*) in Ê. Similarly, ${ď}_{2\overset{\cdot }{G}}\left(U^{\prime },\tilde{V}\right)\neq {ď}_{2\overset{\cdot }{G}}\left(x,y\right)\Rightarrow {ď}_{2\overset{\cdot }{G}}\left(U^{\prime },\tilde{V}\right)+\varsigma_{2}\neq {ď}_{2\overset{\cdot }{G}}\left(x,y\right)+\varsigma_{2}$$\Rightarrow {ď}_{2\overset{\cdot }{G}}\left(U^{\prime },\tilde{V}\right)+\;e_{2}\left(U^{\prime },\tilde{V}\right)\neq {ď}_{2\overset{\cdot }{G}}\left(x,y\right)+\;e_{2}\left(x,y\right)\Rightarrow t{ď}_{2\overset{\cdot }{G}}\left(U^{\prime },\tilde{V}\right)\neq t{ď}_{2\overset{\cdot }{G}}\left(x,y\right)$ for any pair of $\left(U^{\prime },\tilde{V}\right)$ and (*x*, *y*) in Ê and ${ď}_{3\overset{\cdot }{G}}\left(U^{\prime },\tilde{V}\right)\neq {ď}_{3\overset{\cdot }{G}}\left(x,y\right)\Rightarrow {ď}_{3\overset{\cdot }{G}}\left(U^{\prime },\tilde{V}\right)+\varsigma_{3}\neq {ď}_{3\overset{\cdot }{G}}\left(x,y\right)+\varsigma_{3}$$\Rightarrow {ď}_{3\overset{\cdot }{G}}\left(U^{\prime },\tilde{V}\right)+\;e_{3}\left(U^{\prime },\tilde{V}\right)\neq {ď}_{3\overset{\cdot }{G}}\left(x,y\right)+\;e_{3}\left(x,y\right)\Rightarrow t{ď}_{3\overset{\cdot }{G}}\left(U^{\prime },\tilde{V}\right)\neq t{ď}_{3\overset{\cdot }{G}}\left(x,y\right)$ for any pair of $\left(U^{\prime },\tilde{V}\right)$ and (*x*, *y*) in Ê. Therefore, $t{ď}_{\overset{\cdot }{G}}\left(U^{\prime },\tilde{V}\right)\neq t{ď}_{\overset{\cdot }{G}}\left(x,y\right)$. Hence, $\overset{\cdot }{G}$ is strongly ETISVNG.

**Theorem 9 .**

If a connected SVNG $\overset{\cdot }{G}=\left(\tilde{V},\text{Ê}\right)$ is strongly ETISVNG and Ê is a constant function, then $\overset{\cdot }{G}$ is strongly EISVNG.

**Proof**

Suppose that Ê is constant function. Assume that $e_{1}\left(U^{\prime },\tilde{V}\right)=\varsigma_{1}$, $e_{2}\left(U^{\prime },\tilde{V}\right)=\varsigma_{2}$, and $e_{3}\left(U^{\prime },\tilde{V}\right)=\varsigma_{3}$; for all $e_{i}\left(U^{\prime },\tilde{V}\right)\in \text{Ê}$, *ς*_1_, *ς*_2_, and *ς*_3_ are constants. Let $\overset{\cdot }{G}$ be strongly ETISVNG. Then, all pairs of edges are with distinct total degrees. Suppose $\left(U^{\prime },\tilde{V}\right)$ and (*x*, *y*) be any pair of edge in Ê. Then, $t{ď}_{1\overset{\cdot }{G}}\left(U^{\prime },\tilde{V}\right)\neq t{ď}_{1\overset{\cdot }{G}}\left(x,y\right)\Rightarrow {ď}_{1\overset{\cdot }{G}}\left(U^{\prime },\tilde{V}\right)+\;e_{1}\left(U^{\prime },\tilde{V}\right)\neq {ď}_{1\overset{\cdot }{G}}\left(x,y\right)+\;e_{1}\left(x,y\right)$$\Rightarrow {ď}_{1\overset{\cdot }{G}}\left(U^{\prime },\tilde{V}\right)+\varsigma_{1}\neq {ď}_{1\overset{\cdot }{G}}\left(x,y\right)+\varsigma_{1}\Rightarrow {ď}_{1\overset{\cdot }{G}}\left(U^{\prime },\tilde{V}\right)\neq {ď}_{1\overset{\cdot }{G}}\left(x,y\right)$ for any pair of edge $\left(U^{\prime },\tilde{V}\right)$ and (*x*, *y*) in Ê. Similarly, $t{ď}_{2\overset{\cdot }{G}}\left(U^{\prime },\tilde{V}\right)\neq t{ď}_{2\overset{\cdot }{G}}\left(x,y\right)\Rightarrow {ď}_{2\overset{\cdot }{G}}\left(U^{\prime },\tilde{V}\right)+\;e_{2}\left(U^{\prime },\tilde{V}\right)\neq {ď}_{2\overset{\cdot }{G}}\left(x,y\right)+\;e_{2}\left(x,y\right)$$\Rightarrow {ď}_{2\overset{\cdot }{G}}\left(U^{\prime },\tilde{V}\right)+\varsigma_{2}\neq {ď}_{2\overset{\cdot }{G}}\left(x,y\right)+\varsigma_{2}\Rightarrow {ď}_{2\overset{\cdot }{G}}\left(U^{\prime },\tilde{V}\right)\neq {ď}_{2\overset{\cdot }{G}}\left(x,y\right)$ for any pair of edge $\left(U^{\prime },\tilde{V}\right)$ and (*x*, *y*) in Ê and $t{ď}_{3\overset{\cdot }{G}}\left(U^{\prime },\tilde{V}\right)\neq t{ď}_{3\overset{\cdot }{G}}\left(x,y\right)\Rightarrow {ď}_{3\overset{\cdot }{G}}\left(U^{\prime },\tilde{V}\right)+\;e_{3}\left(U^{\prime },\tilde{V}\right)\neq {ď}_{3\overset{\cdot }{G}}\left(x,y\right)+\;e_{3}\left(x,y\right)$$\Rightarrow {ď}_{3\overset{\cdot }{G}}\left(U^{\prime },\tilde{V}\right)+\varsigma_{3}\neq {ď}_{3\overset{\cdot }{G}}\left(x,y\right)+\varsigma_{3}\Rightarrow {ď}_{3\overset{\cdot }{G}}\left(U^{\prime },\tilde{V}\right)\neq {ď}_{3\overset{\cdot }{G}}\left(x,y\right)$ for any pair of edge $\left(U^{\prime },\tilde{V}\right)$ and (*x*, *y*) in Ê. Therefore, ${ď}_{\overset{\cdot }{G}}\left(U^{\prime },\tilde{V}\right)\neq {ď}_{\overset{\cdot }{G}}\left(x,y\right)$. Hence, $\overset{\cdot }{G}$ is strongly EISVNG.

**Theorem 10 .**

If a connected SVNG $\overset{\cdot }{G}=\left(\tilde{V},\text{Ê}\right)$ is strongly EISVNG and Ê is a constant function, then $\overset{\cdot }{G}$ is strongly ISVNG.

**Proof**

Suppose that Ê is a constant function. Assume that $e_{1}\left(U^{\prime },\tilde{V}\right)=\varsigma_{1}$, $e_{2}\left(U^{\prime },\tilde{V}\right)=\varsigma_{2}$, and $e_{3}\left(U^{\prime },\tilde{V}\right)=\varsigma_{3}$; for all $e_{i}\left(U^{\prime },\tilde{V}\right)\in \text{Ê}$, *ς*_1_, *ς*_2_, and *ς*_3_ are constants. Now, assume that $\overset{\cdot }{G}$ is strongly EISVNG. Then, every pair of edges is with distinct degrees. Suppose $\left(U^{\prime },\tilde{V}\right)$ and $\left(\tilde{V},\omega \right)$ be neighboring to the edges with distinct degrees. Then, ${ď}_{1\overset{\cdot }{G}}\left(U^{\prime },\tilde{V}\right)\neq {ď}_{1\overset{\cdot }{G}}\left(\tilde{V},\omega \right)\Rightarrow \;{ď}_{1\overset{\cdot }{G}}\left(U^{\prime }\right)+{ď}_{1\overset{\cdot }{G}}\left(\tilde{V}\right)\;-2\;e_{1}\left(U^{\prime },\tilde{V}\right)\neq {ď}_{1\overset{\cdot }{G}}\left(\tilde{V}\right)+{ď}_{1\overset{\cdot }{G}}\left(\omega \right)$$-2\;e_{1}\left(\tilde{V},\omega \right)\Rightarrow \;{ď}_{1\overset{\cdot }{G}}\left(U^{\prime }\right)+{ď}_{1\overset{\cdot }{G}}\left(\tilde{V}\right)\;-2\varsigma_{1}\neq {ď}_{1\overset{\cdot }{G}}\left(\tilde{V}\right)+{ď}_{1\overset{\cdot }{G}}\left(\omega \right)\;-2\varsigma_{1}$${ď}_{\overset{\cdot }{G}}\left(U^{\prime }\right)\neq {ď}_{1\overset{\cdot }{G}}\left(\omega \right)$. Also, ${ď}_{2\overset{\cdot }{G}}\left(U^{\prime },\tilde{V}\right)\neq {ď}_{2\overset{\cdot }{G}}\left(\tilde{V},\omega \right)\Rightarrow \;{ď}_{2\overset{\cdot }{G}}\left(U^{\prime }\right)+{ď}_{2\overset{\cdot }{G}}\left(\tilde{V}\right)\;-2\;e_{2}\left(U^{\prime },\tilde{V}\right)\neq {ď}_{2\overset{\cdot }{G}}\left(\tilde{V}\right)+{ď}_{2\overset{\cdot }{G}}\left(\omega \right)$$-2\;e_{2}\left(\tilde{V},\omega \right)\Rightarrow \;{ď}_{2\overset{\cdot }{G}}\left(U^{\prime }\right)+{ď}_{2\overset{\cdot }{G}}\left(\tilde{V}\right)\;-2\varsigma_{2}\neq {ď}_{2\overset{\cdot }{G}}\left(\tilde{V}\right)+{ď}_{2\overset{\cdot }{G}}\left(\omega \right)-2\varsigma_{2}$${ď}_{\overset{\cdot }{G}}\left(U^{\prime }\right)\neq {ď}_{1\overset{\cdot }{G}}\left(\tilde{V}\right)$ and ${ď}_{3\overset{\cdot }{G}}\left(U^{\prime },\tilde{V}\right)\neq {ď}_{3\overset{\cdot }{G}}\left(\tilde{V},\omega \right)\Rightarrow \;{ď}_{3\overset{\cdot }{G}}\left(U^{\prime }\right)+{ď}_{3\overset{\cdot }{G}}\left(\tilde{V}\right)-2\;e_{3}\left(U^{\prime },\tilde{V}\right)\neq {ď}_{3\overset{\cdot }{G}}\left(\tilde{V}\right)+{ď}_{3\overset{\cdot }{G}}\left(\omega \right)$$-2e_{3}\left(\tilde{V},\omega \right)\Rightarrow \;{ď}_{3\overset{\cdot }{G}}\left(U^{\prime }\right)+{ď}_{3\overset{\cdot }{G}}\left(\tilde{V}\right)-2\varsigma_{3}\neq {ď}_{3\overset{\cdot }{G}}\left(\tilde{V}\right)+{ď}_{3\overset{\cdot }{G}}\left(\omega \right)-2\varsigma_{3}$${ď}_{\overset{\cdot }{G}}\left(U^{\prime }\right)\neq {ď}_{1\overset{\cdot }{G}}\left(\omega \right)$. Therefore, a vertex $\tilde{V}$ neighbors to the vertices *U*′ and *ω* with different degrees. Hence, $\overset{\cdot }{G}$ is strongly ISVNG. The degree of edge irregularity is now extended to path and cycle of SVNG. It is explained in Section 5.

## 5. Edge Irregularity on a Path and a Cycle in SVNG

**Theorem 11 .**

If a path contains 2*m*(*m* > 1) of vertices in a SVNG $\overset{\cdot }{G}$ and if the degrees of edges membership, indeterminacy, and nonmembership are the same, then $\overset{\cdot }{G}$ is both EISVNG and $\overset{\cdot }{G}$ ETISVNG. However, $\overset{\cdot }{G}$ is not HEISVNG and $\overset{\cdot }{G}$ is not HETSVNG.

**Proof**

**Figure 9:**
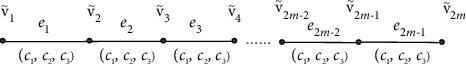
Single-valued neutrosophic graph path.

Suppose that a path contains 2*m*(*m* > 1) of vertices in $\overset{\cdot }{G}$. Let *e*_1_, *e*_2_, *e*_3_,…, *e*_2*m*−1_ be all the edges of $\overset{\cdot }{G}$. If all the grades of membership, indeterminacy, and nonmembership are the same which is *ς*_1_, *ς*_2_, and *ς*_3_, as shown in Figure 9, then ${ď}_{1\overset{\cdot }{G}}\left(\;e_{1}\right)=\varsigma_{1}+2\varsigma_{1}-2\varsigma_{1}=\varsigma_{1},{ď}_{1\overset{\cdot }{G}}\left(\;e_{i}\right)=2\varsigma_{1}+2\varsigma_{1}-2\varsigma_{1}=2\varsigma_{1},i=1,2,\ldots ,2m-2$, ${ď}_{2\overset{\cdot }{G}}\left(\;e_{2m-1}\right)=\varsigma_{1}+2\varsigma_{1}-2\varsigma_{1}=\varsigma_{1},{ď}_{2\overset{\cdot }{G}}\left(\;e_{1}\right)=\varsigma_{2}+2\varsigma_{2}-2\varsigma_{2}=\varsigma_{2},{ď}_{1\overset{\cdot }{G}}\left(\;e_{i}\right)=2\varsigma_{2}+2\varsigma_{2}-2\varsigma_{2}=2\varsigma_{2},i=1,2,\ldots ,2m-2$${ď}_{1\overset{\cdot }{G}}\left(\;e_{2m-1}\right)=\varsigma_{2}+2\varsigma_{2}-2\varsigma_{2}=\varsigma_{2}$, ${ď}_{3\overset{\cdot }{G}}\left(\;e_{1}\right)=\varsigma_{3}+2\varsigma_{3}-2\varsigma_{3}=\varsigma_{3},{ď}_{3\overset{\cdot }{G}}\left(\;e_{i}\right)=2\varsigma_{3}+2\varsigma_{3}-2\varsigma_{3}=2\varsigma_{3},i=1,2,\ldots ,2m-2$, and ${ď}_{3\overset{\cdot }{G}}\left(\;e_{2m-1}\right)=\varsigma_{3}+2\varsigma_{3}-2\varsigma_{3}=\varsigma_{3}$. Note that the neighboring edges of  *e*_2_ are  *e*_1_ and  *e*_3_ with distinct degrees. Hence, $\overset{\cdot }{G}$ is an EISVNG but not HEISVNG. Again, $t{ď}_{1\overset{\cdot }{G}}\left(\;e_{1}\right)=\varsigma_{1}+2\varsigma_{1}-\varsigma_{1}=2\varsigma_{1},{ď}_{1\overset{\cdot }{G}}\left(\;e_{i}\right)=2\varsigma_{1}+2\varsigma_{1}-\varsigma_{1}=3\varsigma_{1},i=1,2,\ldots ,2M-2$, ${ď}_{1\overset{\cdot }{G}}\left(\;e_{2m-1}\right)=\varsigma_{1}+2\varsigma_{1}-\varsigma_{1}=2\varsigma_{1},t{ď}_{2\overset{\cdot }{G}}\left(\;e_{1}\right)=\varsigma_{2}+2\varsigma_{2}-\varsigma_{2}=2\varsigma_{2},{ď}_{2\overset{\cdot }{G}}\left(\;e_{i}\right)=2\varsigma_{2}+2\varsigma_{2}-\varsigma_{2}=3\varsigma_{2},i=1,2,\ldots ,2m-2$, ${ď}_{2\overset{\cdot }{G}}\left(\;e_{2m-1}\right)=\varsigma_{2}+2\varsigma_{2}-\varsigma_{2}=2\varsigma_{2}$, $t{ď}_{3\overset{\cdot }{G}}\left(\;e_{1}\right)=\varsigma_{3}+2\varsigma_{3}-\varsigma_{3}=2\varsigma_{3},{ď}_{3\overset{\cdot }{G}}\left(\;e_{i}\right)=2\varsigma_{3}+2\varsigma_{3}-\varsigma_{3}=3\varsigma_{3},i=1,2,\ldots ,2m-2$, and ${ď}_{3\overset{\cdot }{G}}\left(\;e_{2m-1}\right)=\varsigma_{3}+2\varsigma_{3}-\varsigma_{3}=2\varsigma_{3}$. Likewise, the neighboring edges of  *e*_2_ are  *e*_1_ and  *e*_3_ with different degrees. Hence, $\overset{\cdot }{G}$ is an ETISVNG but not HETISVNG.

**Theorem 12 .**

If a path contains 2*m*(*m* > 1) vertices in a SVNG $\overset{\cdot }{G}$ and the alternating edges' membership, indeterminacy, and nonmembership are the same values, then $\overset{\cdot }{G}$ is both EISVNG and an ETISVNG. However, $\overset{\cdot }{G}$ is not HEISVNG and $\overset{\cdot }{G}$ is not HETSVNG.

**Proof**

Suppose that if a path contains 2*m*(*m* > 1) vertices in a SVNG $\overset{\cdot }{G}$ and the alternating edges' membership, indeterminacy, and nonmembership are the same values, then assume

(5) $$\begin{array}{c} e_{1}\left(e_{i}\right)=\left\{\begin{array}{cc} \varsigma_{1}, & \text{if }i\text{ is odd,}\\ c_{2}, & \text{if }i\text{ is even with }c_{1}\neq c_{2}, \end{array}\right.\\ e_{2}\left(e_{i}\right)=\left\{\begin{array}{cc} \varsigma_{3}, & \text{if }i\text{ is odd,}\\ c_{4}, & \text{if }i\text{ is even with }c_{3}\neq c_{4}, \end{array}\right.\;\\ e_{3}\left(e_{i}\right)=\left\{\begin{array}{cc} \varsigma_{5}, & \text{if }i\text{ is odd,}\\ c_{6}, & \text{if }i\text{ is even with }c_{5}\neq c_{6}. \end{array}\right) \end{array}$$

Then, ${ď}_{1\overset{\cdot }{G}}\left(\;e_{1}\right)=\varsigma_{1}+\varsigma_{1}+\varsigma_{2}-2\varsigma_{1}=\varsigma_{2},{ď}_{1\overset{\cdot }{G}}\left(\;e_{i}\right)=\varsigma_{1}+\varsigma_{2}+\varsigma_{1}+\varsigma_{2}-2\varsigma_{1}=2\varsigma_{2},i=3,5,7,\ldots ,2m-3$, ${ď}_{1\overset{\cdot }{G}}\left(\;e_{i}\right)=\varsigma_{1}+\varsigma_{2}+\varsigma_{1}+\varsigma_{2}-2\varsigma_{2}=2\varsigma_{1},i=2,4,6,\ldots ,2m-2$, ${ď}_{1\overset{\cdot }{G}}\left(\;e_{2m-1}\right)=\varsigma_{1}+\varsigma_{1}+\varsigma_{2}-2\varsigma_{1}=\varsigma_{2}$, ${ď}_{2\overset{\cdot }{G}}\left(\;e_{1}\right)=\varsigma_{3}+\varsigma_{3}+\varsigma_{4}-2\varsigma_{3}=\varsigma_{4},{ď}_{2\overset{\cdot }{G}}\left(\;e_{i}\right)=\varsigma_{3}+\varsigma_{4}+\varsigma_{3}+\varsigma_{4}-2\varsigma_{3}=2\varsigma_{4},i=3,5,7,\ldots ,2m-3$, ${ď}_{2\overset{\cdot }{G}}\left(\;e_{i}\right)=\varsigma_{3}+\varsigma_{4}+\varsigma_{3}+\varsigma_{4}-2\varsigma_{4}=2\varsigma_{3},i=2,4,6,\ldots ,2m-2$, ${ď}_{2\overset{\cdot }{G}}\left(\;e_{2m-1}\right)=\varsigma_{3}+\varsigma_{3}+\varsigma_{4}-2\varsigma_{3}=\varsigma_{4}$, ${ď}_{3\overset{\cdot }{G}}\left(\;e_{1}\right)=\varsigma_{5}+\varsigma_{5}+\varsigma_{6}-2\varsigma_{5}=\varsigma_{6},{ď}_{3\overset{\cdot }{G}}\left(\;e_{i}\right)=\varsigma_{5}+\varsigma_{6}+\varsigma_{5}+\varsigma_{6}-2\varsigma_{5}=2\varsigma_{6},i=3,5,7,\ldots ,2m-3$, ${ď}_{3\overset{\cdot }{G}}\left(\;e_{i}\right)=\varsigma_{5}+\varsigma_{6}+\varsigma_{5}+\varsigma_{6}-2\varsigma_{6}=2\varsigma_{5},i=2,4,6,\ldots ,2m-2$, and ${ď}_{3\overset{\cdot }{G}}\left(\;e_{2m-1}\right)=\varsigma_{5}+\varsigma_{5}+\varsigma_{6}-2\varsigma_{5}=\varsigma_{6}$.

Note that  *e*_2_ and  *e*_3_ neighboring edges have distinct degrees. Hence, $\overset{\cdot }{G}$ is EISVNG but not HEISVNG. Next, $t{ď}_{1\overset{\cdot }{G}}\left(\;e_{1}\right)=\varsigma_{1}+\varsigma_{1}+\varsigma_{2}-\varsigma_{1}=\varsigma_{1}+\varsigma_{2},t{ď}_{1\overset{\cdot }{G}}\left(\;e_{i}\right)=\varsigma_{1}+\varsigma_{2}+\varsigma_{1}+\varsigma_{2}-\varsigma_{1}=\varsigma_{1}+2\varsigma_{2},i=3,5,7,\ldots ,2m-3$, $t{ď}_{1\overset{\cdot }{G}}\left(\;e_{i}\right)=\varsigma_{1}+\varsigma_{2}+\varsigma_{1}+\varsigma_{2}-\varsigma_{2}=2\varsigma_{1}+\varsigma_{2},i=2,4,6,\ldots ,2m-2$, $t{ď}_{1\overset{\cdot }{G}}\left(\;e_{2m-1}\right)=\varsigma_{1}+\varsigma_{1}+\varsigma_{2}-\varsigma_{1}=\varsigma_{1}+\varsigma_{2}$, $t{ď}_{2\overset{\cdot }{G}}\left(\;e_{1}\right)=\varsigma_{3}+\varsigma_{3}+\varsigma_{4}-\varsigma_{3}=\varsigma_{3}+\varsigma_{4},t{ď}_{2\overset{\cdot }{G}}\left(\;e_{i}\right)=\varsigma_{3}+\varsigma_{4}+\varsigma_{3}+\varsigma_{4}-\varsigma_{3}=\varsigma_{3}+2\varsigma_{4},i=3,5,7,\ldots ,2m-3$, $t{ď}_{2\overset{\cdot }{G}}\left(\;e_{i}\right)=\varsigma_{3}+\varsigma_{4}+\varsigma_{3}+\varsigma_{4}-\varsigma_{4}=\varsigma_{4}+2\varsigma_{3},i=2,4,6,\ldots ,2m-2$, $t{ď}_{2\overset{\cdot }{G}}\left(\;e_{2m-1}\right)=\varsigma_{3}+\varsigma_{3}+\varsigma_{4}-\varsigma_{3}=\varsigma_{3}+\varsigma_{4}$, $t{ď}_{3\overset{\cdot }{G}}\left(\;e_{1}\right)=\varsigma_{5}+\varsigma_{5}+\varsigma_{6}-\varsigma_{5}=\varsigma_{5}+\varsigma_{6},{ď}_{3\overset{\cdot }{G}}\left(\;e_{i}\right)=\varsigma_{5}+\varsigma_{6}+\varsigma_{5}+\varsigma_{6}-\varsigma_{5}=\varsigma_{5}+2\varsigma_{6},i=3,5,7,\ldots ,2m-3$, ${ď}_{3\overset{\cdot }{G}}\left(\;e_{i}\right)=\varsigma_{5}+\varsigma_{6}+\varsigma_{5}+\varsigma_{6}-\varsigma_{6}=2\varsigma_{5}+\varsigma_{6},i=2,4,6,\ldots ,2m-2$, and $t{ď}_{3\overset{\cdot }{G}}\left(\;e_{2m-1}\right)=\varsigma_{5}+\varsigma_{5}+\varsigma_{6}-\varsigma_{5}=\varsigma_{5}+\varsigma_{6}$.

Note that  *e*_2_ and  *e*_3_ neighboring edges are with different degrees. Hence, $\overset{\cdot }{G}$ is TEISVNG but not HTEISVNG.

**Theorem 13 .**

If an even cycle has length  2*m* in a SVNG $\overset{\cdot }{G}$ and if the alternating membership of edges, indeterminacy, and nonmembership are the same values, then, $\overset{\cdot }{G}$ is not an EISVNG and not an ETISVNG.

**Proof**

It follows the proof of Theorem 12. Therefore, it is omitted.

## 6. Application

In this section, we utilize the notion of SVNGs to a DM problem. A group of DM problems regarding the “choice of selecting the most significant investment object” is solved to elaborate applications of the suggested notion of SVNGs in a practical scenario that builds on neutrosophic fuzzy preference relations (PFPRs).

### 6.1. Selection of the Most Significant Investment Object

An investor who is a risk fonder likes to put an idle fund into the Shanghai Stock Exchange as a long-term savings. According four companies, ${\underline{z}}_{i}$, (*i*=1,2,3,4) are incredibly hopeful which symbolize four different industries. His resources, i.e., time and energy, are limited to his diplomacy to select largely significant investment object from the available choices. Consequently, he confers his investment adviser ${\bar{e}}_{1}$ and three stock specialists $\;{\bar{e}}_{2}$, ${\bar{e}}_{3}$, and ${\bar{e}}_{4}$. Comparison of four companies with admiration to the likelihood of the growing trend of the stock prices is done by the decision makers and the appraisements of these corporate stocks and presents their favourite information on ${\underline{z}}_{i}$(*i*=1,2,3,4), which is shown by the neutrosophic fuzzy element (NFE) *𝒩*_*ij*_^*k*^  which represents the preferences of experts $\;{\bar{e}}_{k}\left(k=1,2,3,4\right)$ over each pair of stocks. The equivalent NFPRs *R*_*k*_=*𝒩*_*ij*_^*k*^_*n*×*n*_ are shown as follows.

**Figure 10:**
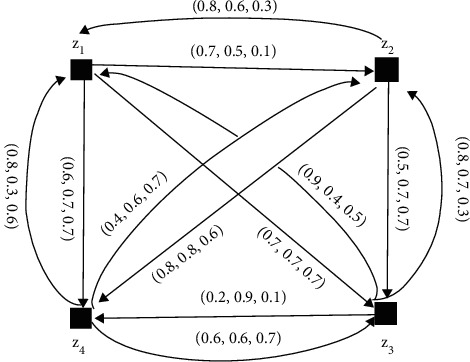
Directed network of the single-valued neutrosophic information for equation (6).

**Figure 11:**
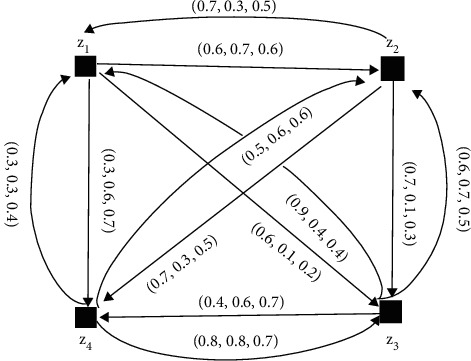
Directed network of the single-valued neutrosophic information for equation (7).

**Figure 12:**
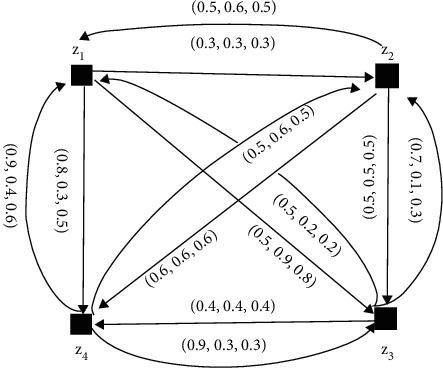
Directed network of the single-valued neutrosophic information for equation (8).

**Figure 13:**
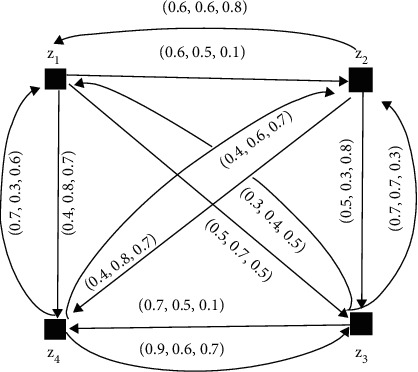
Directed network of the single-valued neutrosophic information for equation (9).

The NFDGs ${\underline{D}}_{i}$ corresponding to NFPRs *R*_*k*_(*k*=1,2,3,4) given in equations (6)–(9) are presented in Figures 10–13, respectively.

Figure 11 represents the SVNDG.

SVNDG is given in Figure 12 and Figure 13.

Next, the single-valued neutrosophic preference relation is given below:

(6) $$\begin{array}{c} R_{1}=\left[\begin{array}{cccc} \left(0.5,0.5,0.5\right) & \left(0.7,0.5,0.1\right) & \left(0.7,0.7,0.7\right) & \left(0.6,0.7,0.7\right)\\ \left(0.8,0.6,0.3\right) & \left(0.5,0.5,0.5\right) & \left(0.5,0.7,0.7\right) & \left(0.8,0.8,0.6\right)\\ \left(0.9,0.4,0.5\right) & \left(0.8,0.7,0.3\right) & \left(0.5,0.5,0.5\right) & \left(0.2,0.9,0.1\right)\\ \left(0.8,0.3,0.6\right) & \left(0.4,0.6,0.7\right) & \left(0.6,0.6,0.7\right) & \left(0.5,0.5,0.5\right) \end{array}\right]. \end{array}$$

Equation (6) is a single-valued neutrosophic preference relation (SVNPR) of the investment advisor:

(7) $$\begin{array}{c} R_{2}=\left[\begin{array}{cccc} \left(0.5,0.5,0.5\right) & \left(0.6,0.7,0.6\right) & \left(0.6,0.1,0.2\right) & \left(0.3,0.6,0.7\right)\\ \left(0.5,0.5,0.5\right) & \left(0.5,0.5,0.5\right) & \left(0.7,0.1,0.3\right) & \left(0.7,0.3,0.5\right)\\ \left(0.9,0.4,0.4\right) & \left(0.6,0.7,0.5\right) & \left(0.5,0.5,0.5\right) & \left(0.4,0.6,0.7\right)\\ \left(0.3,0.3,0.4\right) & \left(0.5,0.6,0.6\right) & \left(0.8,0.8,0.7\right) & \left(0.5,0.5,0.5\right) \end{array}\right], \end{array}$$

(8) $$\begin{array}{c} R_{3}=\left[\begin{array}{cccc} \left(0.5,0.5,0.5\right) & \left(0.3,0.3,0.3\right) & \left(0.5,0.9,0.8\right) & \left(0.9,0.4,0.6\right)\\ \left(0.5,0.6,0.5\right) & \left(0.5,0.5,0.5\right) & \left(0.5,0.5,0.5\right) & \left(0.5,0.6,0.5\right)\\ \left(0.5,0.2,0.2\right) & \left(0.7,0.1,0.3\right) & \left(0.5,0.5,0.5\right) & \left(0.4,0.4,0.4\right)\\ \left(0.8,0.3,0.5\right) & \left(0.6,0.6,0.6\right) & \left(0.9,0.3,0.3\right) & \left(0.5,0.5,0.5\right) \end{array}\right], \end{array}$$

(9) $$\begin{array}{c} R_{4}=\left[\begin{array}{cccc} \left(0.5,0.5,0.5\right) & \left(0.6,0.5,0.1\right) & \left(0.5,0.7,0.5\right) & \left(0.4,0.8,0.7\right)\\ \left(0.6,0.6,0.8\right) & \left(0.5,0.5,0.5\right) & \left(0.5,0.3,0.8\right) & \left(0.5,0.8,0.6\right)\\ \left(0.3,0.4,0.5\right) & \left(0.7,0.7,0.3\right) & \left(0.5,0.5,0.5\right) & \left(0.7,0.5,0.1\right)\\ \left(0.7,0.3,0.6\right) & \left(0.4,0.6,0.7\right) & \left(0.9,0.6,0.7\right) & \left(0.5,0.5,0.5\right) \end{array}\right]. \end{array}$$

SVNPR of the first, second, and third stock expert are given in (7), (8), and (9), respectively.

Collect all *𝒩*_*ij*_^*k*^(*j*=1,2,3,…, *n*) consistent to the alternative ${\underline{Z}}_{i}$ to find the SVNNs *𝒩*_*i*_^*k*^ of the alternative ${\underline{Z}}_{i}$ over all the other alternatives for the expert ${\tilde{e}}_{k}$ by using and SVN averaging (SVNA) operator:

(10) $$\begin{array}{c} \text{SVNA}=N_{ij}^{k}\left(j=1,2,3,\ldots ,n\right)=\left(1-\prod_{j=1}^{n}{\left(1-{\underset{⌢}{T}}_{ij}\right)}^{1/n},{\left(\prod_{j=1}^{n}{\underset{⌢}{I}}_{ij}\right)}^{1/n},{\left(\prod_{j=1}^{n}{\dot{F}}_{ij}\right)}^{1/n}\right),\;i=1,2,\ldots ,n. \end{array}$$

The aggregation results of the experts *N*_*k*_(*k*=1,2,3,4) are as follows:

(11) $$\begin{array}{c} {\tilde{e}}_{1}:N_{1}^{\left(1\right)}=\left(0.9999320,0.000479,0.0000957\right),N_{2}^{\left(1\right)}=\left(0.99990938,0.0006563,0.0002461\right),\\ N_{3}^{\left(1\right)}=\left(0.99996875,0.0004922,0.0000293\right),N_{4}^{\left(1\right)}=\left(0.99990625,0.0002109,0.0005742\right),\\ {\tilde{e}}_{2}:N_{1}^{\left(2\right)}=\left(0.99995742,0.0000820,0.0001641\right),N_{2}^{\left(2\right)}=\left(0.999912109,0.0000293,0.0001465\right),\\ N_{3}^{\left(2\right)}=\left(0.99995325,0.0003281,0.0002734\right),N_{4}^{\left(2\right)}=\left(0.999863281,0.0002813,0.0003281\right),\\ {\tilde{e}}_{3}:N_{1}^{\left(3\right)}=\left(0.999931641,0.0002109,0.0002813\right),N_{2}^{\left(3\right)}=\left(0.999975859,0.0003516,0.0002441\right),\\ N_{3}^{\left(3\right)}=\left(0.9999824219,0.0000156,0.0000469\right),N_{4}^{\left(3\right)}=\left(0.99998437,0.0001055,0.0001758\right),\\ {\tilde{e}}_{4}:N_{1}^{\left(4\right)}=\left(0.999765625,0.0005409,0.000684\right),\;N_{2}^{\left(4\right)}=\left(0.999804688,\;0.0002813,\;0.0007500\right),\\ N_{3}^{\left(4\right)}=\left(0.999876953,0.0002734,0.0000293\right),N_{4}^{\left(4\right)}=\left(0.999964844,0.0002109,0.0005742\right). \end{array}$$

Now, to find the weight of the experts, for this first, we have to find SVN hamming distance between two SVNSs:

(12) $$\begin{array}{c} \underline{D}\left(N_{1},N_{2}\right)=\frac{1}{3n}\overset{n}{\underset{j=1}{\sum }}\left|{\underline{T}}_{N_{1}}\left({\underline{z}}_{i}\right)-{\underline{T}}_{N_{2}}\left({\underline{z}}_{j}\right)\right|+\left|{\underset{⌢}{I}}_{N_{1}}\left({\underline{z}}_{i}\right)-{\underset{⌢}{I}}_{N_{2}}\left({\underline{z}}_{j}\right)\right|+\left|{\dot{F}}_{N_{1}}\left({\underline{z}}_{i}\right)-{\dot{F}}_{N_{2}}\left({\underline{z}}_{j}\right)\right|. \end{array}$$

Next, determine $\underset{˙}{d}\left({𝒩}_{ij}^{l},{𝒩}_{ij}^{k}\right)$, *i*, *j*=1,2,3,4and *l*,  *k*=1,2,3,4, and find the difference matrix ${\underline{D}}_{lk}=\underline{d}{\left({𝒩}_{ij}^{l},{𝒩}_{ij}^{k}\right)}_{n\times n}$ as follows:

(13) $$\begin{array}{c} {\underline{D}}_{12}={\underline{D}}_{21}=\left[\begin{array}{cccc} 0 & 0.266667 & 0.4 & 0.133333\\ 0.2 & 0 & 0.4 & 0.233333\\ 0.0333333 & 0.133333 & 0 & 0.366667\\ 0.23333 & 0.0666667 & 0.133333 & 0 \end{array}\right],\\ {\underline{D}}_{13}={\underline{D}}_{31}=\left[\begin{array}{cccc} 0 & 0.266667 & 0.166667 & 0.233333\\ 0.1666666667 & 0 & 0.1333333 & 0.2\\ 0.3 & 0.233333 & 0 & 0.3333333\\ 0.0333333 & 0.1 & 0.333333 & 0 \end{array}\right],\\ {\underline{D}}_{14}={\underline{D}}_{41}=\left[\begin{array}{cccc} 0 & 0.033333 & 0.1333333 & 0.1\\ 0.1666666667 & 0 & 0.1666667 & 0.1\\ 0.2 & 0.033333 & 0 & 0.3\\ 0.0333333 & 0 & 0.1 & 0 \end{array}\right],\\ {\underline{D}}_{23}={\underline{D}}_{32}=\left[\begin{array}{cccc} 0 & 0.333333 & 0.5 & 0.3\\ 0.03333333 & 0 & 0.2666667 & 0.166667\\ 0.26666667 & 0.3 & 0 & 0.16666667\\ 0.2 & 0.033333 & 0.333333 & 0 \end{array}\right],\\ {\underline{D}}_{24}={\underline{D}}_{42}=\left[\begin{array}{cccc} 0 & 0.233333 & 0.33333 & 0.1\\ 0.1666666667 & 0 & 0.3 & 0.266667\\ 0.23333333 & 0.1 & 0 & 0.3333333\\ 0.2 & 0.066667 & 0.1 & 0 \end{array}\right],\\ {\underline{D}}_{34}={\underline{D}}_{43}=\left[\begin{array}{cccc} 0 & 0.233333 & 0.166667 & 0.333333\\ 0.133333333 & 0 & 0.166667 & 0.1\\ 0.23333333 & 0.2 & 0 & 0.2333333\\ 0.06666667 & 0.1 & 0.23333333 & 0 \end{array}\right],\\ {\underline{D}}_{11}={\underline{D}}_{22}={\underline{D}}_{33}={\underline{D}}_{4}=\left[\begin{array}{cccc} 0 & 0 & 0 & 0\\ 0 & 0 & 0 & 0\\ 0 & 0 & 0 & 0\\ 0 & 0 & 0 & 0 \end{array}\right]. \end{array}$$

Now, find the average values of the different matrices by using the following equation:

(14) $$\begin{array}{c} {\underset{¯}{d}}_{lk}=\overset{n}{\underset{i=1}{\sum }}\overset{n}{\underset{j=1}{\sum }}{\underset{¯}{d}}_{ij}^{\left(lk\right)},\\ {\underline{d}}_{12}={\underline{d}}_{21}=\frac{2.599999363}{16}=0.1625,\\ {\underline{d}}_{13}={\underline{d}}_{31}=\frac{2.799993903}{16}=0.17499,\\ {\underline{d}}_{14}={\underline{d}}_{41}=\frac{1.36666665967}{16}=0.08541,\\ {\underline{d}}_{32}={\underline{d}}_{23}=\frac{2.900000267}{16}=0.181250,\\ {\underline{d}}_{24}={\underline{d}}_{42}=\frac{2.43333}{16}=0.1520831,\\ {\underline{d}}_{34}={\underline{d}}_{43}=\frac{2.2}{16}=0.1375. \end{array}$$

Next, find the deviation of the expert ${\underline{d}}_{1}$ from the remaining experts by using ${\underline{d}}_{l}=\sum_{k=1,k\neq l}^{n}{\underline{d}}_{ij}^{\left(lk\right)}$:

(15) $$\begin{array}{c} {\underline{d}}_{1}=0.4229,\\ {\underline{d}}_{2}=0.495831,\\ {\underline{d}}_{3}=0.49374,\\ {\underline{d}}_{4}=0.37499931. \end{array}$$

To find the weight of the expert, we use $w_{l}={\left({\underline{d}}_{l}\right)}^{-1}/\sum_{l=1}^{s}{\left({\underline{d}}_{l}\right)}^{-1}$, *l*=1,2,…, *s*:

(16) $$\begin{array}{c} w_{1}=0.26,\\ w_{2}=0.22,\\ w_{3}=0.22,\\ w_{4}=0.29. \end{array}$$

Now, we use the SVNWA operator to find the collective SVNNs *𝒩*_*i*_=SVNWA(*𝒩*_*i*_^(1)^, *𝒩*_*i*_^(2)^,…, *𝒩*_*i*_^(*s*)^) of the company ${\underline{z}}_{i}$ over all the other companies. That is,

(17) $$\begin{array}{c} N_{1}=\left(0.9991056,0.00030424,0.00026506\right),\\ N_{2}=\left(0.9998461,0.000242967,0.000329008\right),\\ N_{3}=\left(0.9999175,0.00019171,0.0005896\right),\\ N_{4}=\left(0.99994228,0.00020948,0.00042161\right). \end{array}$$

To find the rank of all the companies ${\underline{z}}_{i}\left(i=1,2,3,4\right)$, we use Definition 4 of SVNNs score function. Therefore, the values of *S*(*𝒩*_*i*_)(*i*=1,2,3,4) are

(18) $$\begin{array}{c} S\left(N_{1}\right)=0.9995121,\\ S\left(N_{2}\right)=0.99975804,\\ S\left(N_{3}\right)=0.9998889,\\ S\left(N_{4}\right)=0.999770397. \end{array}$$

Then, $\;{\underline{z}}_{3}≻{\underline{z}}_{4}≻{\underline{z}}_{2}≻{\underline{z}}_{1}$. Hence, the ideal choice is ${\underline{z}}_{3}.$

Below is the Algorithm 1 that is purposely used for solving the multicriteria DM problem.

In Section 6.2, we also present the second example to illustrate the proposed graphs.

### 6.2. Selection of the Subjects at Higher Studies

Students of this secondary age have many career choices. Apart from some courses that are chosen mostly, other choices are also the best choices until any single student utilizes enough scheming and enough core interest to subject/career. Interest along with sound preparedness aids in achieving capabilities in any area of work we selected. First choice in career selection is made and required after the secondary pretertiary education of students. At this time, enough information with respect to their interest has to be given. In this part, based on the survey conducted among random sample of 100 students of class *x*, the percentage of students with interest, neutral, and disinterest towards a particular subject and pair of subjects that they have studied till class *x* is calculated and tabulated. SVNG is employed as a device relying on this data as it involves degree of membership (interest of percentage of students to a subject or pair of subjects) and the degree of indeterminacy (neutral percentage of students to a subject or pair of subjects) and the degree of nonmembership (disinterest of percentage of students to a subject or pair of subjects). By employing SVNG, the best combination of subjects can be analysed such as the class that has subjects which can achieve excellent academics performance of many students.

Let *S* = {English (E), Language (L), Maths (M), Science (S), Social Science (SS)} be the collection of vertices.  Table 1 explains the percentage of students with interest, neutral, and disinterest towards a subject.

Table 2 displays the percentages of students with interest, neutral. and disinterest towards pairs of subjects.

**Figure 14:**
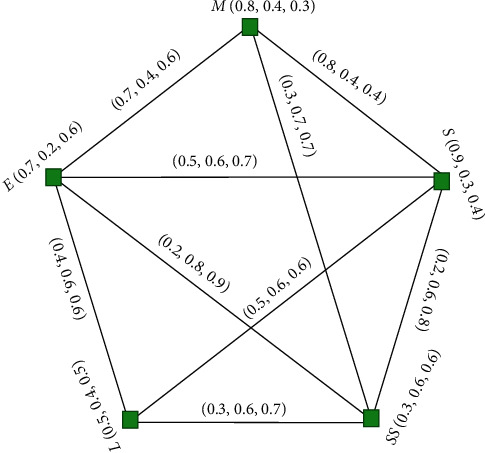
Single-valued neutrosophic graph used for percentage.

Figure 14 is the graph used for all vertices; the degree of membership indicates percentage of students who have interest for a particular subject, the grade of indeterminacy indicates percentage of students who have neutral for a particular subject, and grade of nonmembership is the percentage of students who have disinterests for subject from a random sample of 100 students of class *x* selected for survey. Also, membership, indeterminacy, and nonmembership grades of edges of the graph show the likes, neutral, and dislikes of the students to study the combination of any two subjects at the higher secondary level. From the graph, the edge (E, SS) with high degree of nonmembership shows that the majority of the students do not like to study the combination of English and social science and the edge; (M, S), having high degree of membership, shows majority of the students have zeal to study the combination of maths and science. There is also no interest, neutral, and disinterest to study the combination of language and maths which indicates the subjects that are not required to be combined. Thus, a high or low level of membership of any edge demonstrates the high and low proportion for the combination of the subjects at higher studies.

This easy analysis shows that SVNG can be employed in decision-making situations for all practical and everyday problems. Additional utilization in artificial intelligence and decision-making situations can be examined.

### 6.3. Comparative Study

The suggested novel approach is important because the novel approach can resolve the issues which are present in the environment of IFSs along with FSs. We examine two examples, at present, containing data in the form of IFNs or FNs.

By supposing the decision matrix in which data are presented in the form of IFNs,

(19) $$\begin{array}{c} \dot{R}={\left({\dot{r}}_{ij}\right)}_{4\times 4}=\left(\begin{array}{cccc} \left(0.5\right), & \left(0.3\right), & \left(0.7\right), & \left(0.6\right),\\ \left(0.3\right), & \left(0.5\right), & \left(0.7\right), & \left(0.6\right),\\ \left(0.4\right), & \left(0.2\right), & \left(0.5\right), & \left(0.7\right),\\ \left(0.3\right), & \left(0.6\right), & \left(0.4\right), & \left(0.5\right). \end{array}\right) \end{array}$$

At present, this sort of data can be simply analysed employing the SVNWA and SVNWG operators by assuming $\underset{⌢}{I}=\dot{F}=0.$

In addition, if the information is in the form of IFNs, then the decision matrix is written as

(20) $$\begin{array}{c} \dot{R}={\left({\dot{r}}_{ij}\right)}_{4\times 4}=\left(\begin{array}{cccc} \left(0.5,0.5\right), & \left(0.3,0.6\right), & \left(0.1,0.6\right), & \left(0.5,0.2\right),\\ \left(0.3,0.3\right), & \left(0.5,0.5\right), & \left(0.5,0.2\right), & \left(0.3,0.5\right),\\ \left(0.4,0.4\right), & \left(0.2,0.6\right), & \left(0.5,0.5\right), & \left(0.3,0.6\right),\\ \left(0.1,0.4\right), & \left(0.3,0.5\right), & \left(0.6,0.2\right), & \left(0.5,0.5\right). \end{array}\right) \end{array}$$

After that, the suggested method can also be employed by applying geometric and averaging aggregation operators of IFSs, as described in Definition 6. Diversely, the aggregation methods of FSs or IFSs could not be utilized to the information of SVNSs because of their restricted structures. From this, the significance of new introduced approach is as follows: the imperativeness and importance of the proposed method is that the novel method is capable of solving the problems in the environment of Pythagorean fuzzy set and IFSs.

An overview of the comparative study is represented through Table 3. As a result, it is observed that the fuzzy set is not applicable in certain situations, whereas the intuitionistic fuzzy set also has some limitations. Particularly, the absence of neutral degree and strict constraints on the selection of degrees limits the ability of a decision maker to make perfect decisions. Thus, it leaves the ground open for the neutrosophic set which ticks all the compartments and wins the match of comparison. The advantages of the proposed framework are as follows: (i) talks about three degrees, (ii) selection of values for the degrees does not limit the decision maker, and (iii) each of the degrees can be independently dealt.

## 7. Conclusion

In this article, some new types of SVNGs were introduced. Moreover, this article also explored some graphical ideas which were well supported by appropriate examples. We also developed the degrees such as irregular SVNG, edge irregular neutrosophic graph, and degree of neutrosophic graph under some conditions and elaborated these via examples. Two real-life applications of SVNGs are discussed where the proposed concept is utilized. Additionally, the proposed concept is utilized in two DM problems. In the first problem, we used the weighted averaging and weighted geometric aggregation operators to select the best company among different companies. The other problem was the selection of the best combination of subjects which was solved by ideas of edges in SVNGs. In addition, the advantages of the proposed work were highlighted by establishing a comparative study which includes the choice of three independent degrees without any constraints. In future, more of the DM algorithms will be discussed in contrast to the existing ones.

## Figures and Tables

**Algorithm 1:**
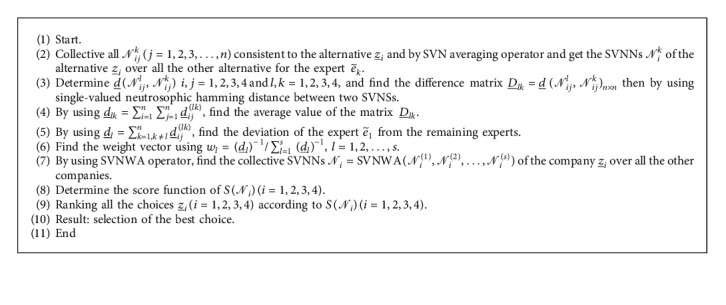
A distinct set of alternative $Z=\left\{{\underline{z}}_{1},\;{\underline{z}}_{2},\;\ldots ,\;{\underline{z}}_{n}\right\}$, set of expert $\tilde{e}=\left\{{\tilde{e}}_{1},\;{\tilde{e}}_{2},\ldots ,{\tilde{e}}_{n}\right\}$, and structure of SVNR *R*_*k*_=(*𝒩*_*ij*_^*k*^)_*n*×*n*_ for each expert.

**Table 1:** Subjects and their degrees.

Subject/subject combination	Interest percentage	Neutral percentage	Disinterest percentage
*E*	0.7	0.2	0.6
*L*	0.5	0.4	0.5
*M*	0.8	0.4	0.3
*S*	0.9	0.3	0.4
*SS*	0.3	0.6	0.6

**Table 2:** Combine subjects and their degrees.

Subject/subject combination	Interest percentage	Neutral percentage	Disinterest percentage
*E* − *M*	0.7	0.4	0.6
*E* − *L*	0.4	0.6	0.6
*E* − *S*	0.5	0.6	0.7
*E* − *SS*	0.2	0.8	0.9
*L* − *M*	0	0	0
*L* − *S*	0.5	0.6	0.6
*L* − *SS*	0.3	0.7	0.6
*M* − *S*	0.8	0.4	0.4
*M* − *SS*	0.3	0.7	0.7
*S* − *SS*	0.2	0.6	0.8

**Table 3:** Summary of the comparison.

Framework	Association degree	Neutral degree	Nonassociation degree	Constraints	Independency
Fuzzy set	Yes	No	No	N/A	N/A
Intuitionistic fuzzy set	Yes	No	Yes	Yes	None
Neutrosophic set	Yes	Yes	Yes	None	Yes

## Data Availability

Data sharing is not applicable to this article as no datasets were generated or analysed during the current study.

## References

[1] Zadeh L. A. (1965). Fuzzy sets. *Information and Control*.

[2] Atanassov K. T. (1986). Intuitionistic fuzzy sets. *Fuzzy Sets and Systems*.

[3] Smarandache F. (2015). Refined literal indeterminacy and the multiplication law of sub-indeterminacies. *Neutrosophic Sets and Systems*.

[4] Wang H., Smarandache F., Zhang Y. Q., Sunderraman R. (2010). Single-valued neutrosophic sets. *Multisspace and Multistruct*.

[5] Shah N. (2016). *Neutrosophic sets and systems*.

[6] Ansari A. Q., Biswas R., Aggarwal S. Neutrosophication of Fuzzy Models.

[7] Ansari A. Q., Biswas R., Aggarwal S. Extension to fuzzy logic representation: moving towards neutrosophic logic - a new laboratory rat.

[8] Majumdar P. (2015). Neutrosophic sets and its applications to decision making, adaptation, learning, and optimization. *Computational Intelligence for Big Data Analysis*.

[9] Majumdar P., Samanta S. K. (2014). On similarity and entropy of neutrosophic sets. *Journal of Intelligent and Fuzzy Systems*.

[10] Nasir A., Jan N., Yang M.-S., Khan S. U. (2021). Complex T-spherical fuzzy relations with their applications in economic relationships and international trades. *IEEE Access*.

[11] Ye J. (2013). Multicriteria decision-making method using the correlation coefficient under single-valued neutrosophic environment. *International Journal of General Systems*.

[12] Ye J. (2014). Improved correlation coefficients of single valued neutrosophic sets and interval neutrosophic sets for multiple attribute decision making. *Journal of Intelligent and Fuzzy Systems*.

[13] Rosenfeld A. (1975). FUZZY GRAPHS††The support of the Office of Computing Activities, National Science Foundation, under Grant GJ-32258X, is gratefully acknowledged, as is the help of Shelly Rowe in preparing this paper. *Fuzzy Sets and their Applications to Cognitive and Decision Processes, Fuzzy Graphs, Fuzzy Sets and Their Applications*.

[14] Parvathi R., Karunambigai M. G. (2006). Intuitionistic fuzzy graphs. *Computational Intelligence, Theory and Applications*.

[15] Bhattacharya P. (1987). Some remarks on fuzzy graphs. *Pattern Recognition Letters*.

[16] Nagoor Gani A., Latha S. R. (2012). On irregular fuzzy graphs. *Applied Mathematical Sciences*.

[17] Davvaz B., Jan N., Mahmood T., Ullah K. (2019). Intuitionistic fuzzy graphs of nth type with applications. *Journal of Intelligent and Fuzzy Systems*.

[18] Mahapatra R., Samanta S., Pal M. (2021). Generalized neutrosophic planar graphs and its application. *Journal of Applied Mathematics and Computing*.

[19] Kalaiarasi K., Divya R. (2021). Shortest path on interval-valued nether trapezoidal neutrosophic fuzzy graphs. *Materials Today Proceedings*.

[20] Nasir A., Jan N., Gumaei A., Khan S. U., Albogamy F. R. (2021). Cybersecurity against the loopholes in industrial control systems using interval-valued complex intuitionistic fuzzy relations. *Applied Sciences*.

[21] Ha S., Liu H., Li S., Liu A. (2019). Backstepping-based adaptive fuzzy synchronization control for a class of fractional-order chaotic systems with input saturation. *International Journal of Fuzzy Systems*.

[22] Mohanta K., Dey A., Pal A. (2021). A note on different types of product of neutrosophic graphs. *Complex & Intelligent Systems*.

[23] Broumi S., Talea M., Bakali A., Smarandache F. (2016). Single-valued neutrosophic graphs. *Journal of New Theory*.

[24] Ramya R., Vinothkumar N., Karuppasamy E. (2021). Complementary domination in Single valued neutrosophic graphs. *Materials Today Proceedings*.

[25] Naz S., Rashmanlou H., Malik M. A. (2017). Operations on single valued neutrosophic graphs with application. *Journal of Intelligent and Fuzzy Systems*.

[26] Abu Saleem M. (2021). A neutrosophic folding and a neutrosophic retraction on a single-valued neutrosophic graph. *Journal of Intelligent & Fuzzy Systems Preprint*.

[27] Lu Z., Ye J. (2017). Single-valued neutrosophic hybrid arithmetic and geometric aggregation operators and their decision-making method. *Information*.

[28] Shahzadi G., Akram M., Saeid A. B. (2017). An application of single-valued neutrosophic sets in medical diagnosis. *Neutrosophic Sets and Systems*.

